# Synergistic activity of simvastatin and irinotecan chemotherapy against glioblastoma converges on TGF-β signaling

**DOI:** 10.1007/s11060-025-05089-8

**Published:** 2025-05-28

**Authors:** Niket Yadav, Aizhen Xiao, Qing Zhong, Pankaj Kumar, Guruprasad Konduru, William Hart, Matthew Lazzara, Benjamin Purow

**Affiliations:** 1 Department of Neurology, University of Virginia School of Medicine, Charlottesville, VA 22903 USA; 2 Medical Scientist Training Program (MSTP), University of Virginia School of Medicine, Charlottesville, VA 22903 USA; 3 Molecular and Cellular Basis of Disease (MCBD) Graduate Program, Department of Pathology, University of Virginia School of Medicine, Charlottesville, VA 22903 USA; 4 Department of Biomedical Engineering, University of Virginia, Charlottesville, VA 22903 USA; 5 Department of Chemical Engineering, University of Virginia, Charlottesville, VA 22903 USA; 6 Department of Biochemistry and Molecular Genetics, University of Virginia, Charlottesville, VA 22903 USA; 7 Bioinformatics Core, University of Virginia, Charlottesville, VA 22903 USA

**Keywords:** Glioblastoma (GBM), Synergy, Simvastatin, Irinotecan, TGF-β, Apoptosis, Autophagy

## Abstract

**Purpose:**

This study investigates the synergistic therapeutic potential of a novel combination of the repurposed drug simvastatin with irinotecan chemotherapy towards glioblastoma (GBM) and the underlying molecular mechanisms.

**Methods:**

In vitro efficacy of simvastatin and irinotecan alone and in combination against diverse GBM lines (U251MG, G34, SB28) was assessed using mechanistically distinct cell viability assays. RNA-Sequencing was performed to uncover the top pathways and genes affected by these drugs, followed by validation of promising pathways (TGF-β signaling and cell death) using targeted phosphoproteomics and in vitro genetic manipulation and functional assays.

**Results:**

We observed robust in vitro synergy at nanomolar concentrations between simvastatin and irinotecan across diverse GBM lines. Notably, irinotecan alone and in combination with simvastatin reduced mRNA expression of TGF-β family members. Targeted phosphoproteomics and functional experiments further showed significant inhibition of TGF-β signaling with both treatment types. Additionally, a role for apoptosis and enrichment of caspase-independent cell death pathways (autophagy, ferroptosis) as well as immunological (interferons, complement, inflammatory responses, TNF-α) and oncogenic (K-RAS/ERK) signaling pathways were observed with the combination treatment.

**Conclusions:**

Besides the first detailed demonstration of a robust synergy between simvastatin and irinotecan against GBM lines, this study shows for the first time that both irinotecan and the combination treatment converge on inhibition of TGF-β signaling. This is notable given the lack of TGF-β inhibitors in the clinic. Collectively, this study provides preclinical data suggesting this novel drug combination be tested in patients with GBM and TGF-β driven cancers.

**Supplementary Information:**

The online version contains supplementary material available at 10.1007/s11060-025-05089-8.

## Introduction

Glioblastoma (GBM) is the most aggressive primary brain tumor, with a median survival of 15–18 months and annual mortality of 13,000 lives in the U.S. [1–5]. Conventional therapies for GBM (surgical resection, radiation, and temozolomide chemotherapy) have limited success, due in part to numerous GBM resistance mechanisms and the unique milieu of the brain. Years-long development of new therapies presents another barrier to advancing GBM care. This supports getting the most from existing agents, including repurposing drugs for other indications—enabling identification of new strategies that can advance directly to clinical trials [6].

In this vein, our group has recently reported single-agent activity of the FDA-approved, commonly prescribed, well-tolerated cholesterol-lowering drug simvastatin against models of human GBM, mechanistically through inhibition of the pro-oncogenic TGF-β signaling pathway [7]. Notably, simvastatin has been suggested to have one of the highest blood–brain barrier (BBB) permeabilities among the statin drug class given its lipophilicity, and modest permeability overall [8–11]. Furthermore, recent studies have suggested that cholesterol-lowering statin drugs may increase the efficacy of anti-cancer drugs, including topoisomerase I inhibitors, in various cancer models but this is poorly understood mechanistically [1, 12–17]. Based on indications from the CellMiner database, we noted potential for drug synergy combining topoisomerase I inhibitors with other agents. Among clinically available topoisomerase I inhibitors, irinotecan (CPT-11) was the top candidate for combination with simvastatin, given its prior clinical use in GBM, modest side-effect profile, and its reported modest BBB permeability [18, 19].

We therefore investigated whether combining simvastatin and irinotecan is synergistic against GBM in vitro and sought underlying signaling mechanisms. Our findings indicate synergistic in vitro efficacy of combining simvastatin and irinotecan against diverse GBM lines at nanomolar concentrations, as well as mechanistic convergence on the TGF-β pathway—suggesting a novel therapeutic option for patients with GBM and other TGF-β driven cancers.

Importantly, TGF-β signaling has been reported to play a prominent role in GBM pathogenesis and cancer in general. TGF-β appears to be pro-tumorigenic in GBM and other cancers through tumor-intrinsic stimulation of cancer survival, epithelial-to-mesenchymal transition (EMT) or analogous processes, treatment resistance, invasiveness, and tumor-extrinsic immunosuppression mechanisms in the tumor microenvironment (TME) that foster cancer immune evasion [20–27]. Notably, there are no TGF-β inhibitors available in the clinic, underscoring the importance of identifying rapidly translatable candidates against this prominent pro-oncogenic pathway [28].

## Materials and methods

### Drugs and reagents

Simvastatin and irinotecan were purchased from Sigma. The cell death inhibitors (Z-VAD-FMK, necrostatin-1, and ferrostatin-1) and TGF-β inhibitors (galunisertib, and LY2109761) were purchased from SelleckChem. Chloroquine and TGF-β1 were purchased from Sigma. SBE4-luc plasmid was purchased from Addgene and expanded and purified using MaxiPrep plasmid isolation kits (Qiagen). BFP and Smad3CA plasmids were generous gifts from Dr. Keisuke Kaji (University of Edinburgh).

### Cell lines and culture conditions

The human GBM cell line U251MG was obtained from American Type Culture Collection (ATCC). The genetically-derived murine cell line SB28 (created by John Ohlfest) was obtained from the DSMZ repository. The primary, patient-derived glioblastoma stem-like cell (GSC) line G34 was obtained from Jakub Godlewski (Harvard University Medical School) and Ichiro Nakano (University of Alabama), and has been previously characterized [29, 30]. U251MG and SB28 lines were cultured in DMEM high-glucose medium (Gibco) supplemented with 10% FBS (Avantor® seradigm via VWR) and 1% penicillin/streptomycin (Gibco). G34 (GSC) cells were cultured in serum-free Neurobasal medium (Gibco) supplemented with N2 and B27 (without vitamin A; Gibco, 0.5X final concentration) and with human recombinant bFGF and EGF (25 ng/mL final concentration each, R&D Systems) and 1% penicillin/streptomycin (Gibco).

### In vitro cell viability assays on GBM/GSC lines

The alamarBlue assay kit (ThermoFisher Scientific), CyQuant Direct Cell Proliferation assay kit (ThermoFisher Scientific), and/or crystal violet (Sigma) staining-based microscopy assay were used for assessment of in vitro cell viability of GBM/GSC cells per manufacturer’s recommendations. 1000 cells/well U251MG or 300 cells/well for SB28 were seeded in 96-well tissue culture treated plates, then drug added the next day. Cells were treated for five days, then assayed as above using a SpectraMax ID3 Plate Reader. Crystal violet-stained U251MG and SB28 whole wells were imaged (lowest magnification setting) with a Leica Thunder microscope (Leica Microsystems) using the “spiral” feature and the automated cell counting feature was employed to count the number of stained nuclei per unit area using the built-in nuclei thresholding feature for image segmentation (U251MG line). For G34 GSCs, 3000 cells (neurospheres were dissociated with Versene solution) were seeded in manually pre-coated 96-well tissue culture treated plates (0.01% Poly-L-Ornithine supplemented with 10 ug/mL laminin) to facilitate maximum adherence and treated for five days before alamarBlue or CyQuant assay. DMSO vehicle concentration was equalized between all treatment groups in all experiments across all lines to control for any baseline vehicle toxicity.

### Primary glioblastoma stem cell tumorsphere formation assays

1000 G34 single cells were seeded per well in uncoated, non-tissue culture treated ultra-low attachment 24-well plates in serum-free complete Neurobasal medium (medium composition stated previously) to facilitate 3D tumorsphere/spheroid growth. A few hours after seeding, cells were treated with drug working solution and then allowed to incubate for approximately two weeks until formation of distinct, large tumorspheres in the DMSO (vehicle)-treated control group. Large spheres were defined as approximately 300 µm with a dark core and medium spheres were defined as approximately 150 µm based on preliminary experiments and measurement in ImageJ software. On the day of assay, plates were first allowed to equilibrate at room temperature for a few minutes to allow the majority of tumorspheres to collect near the center of wells, and then each group was imaged using an EVOS Light Microscope. Subsequently, the number of large and medium tumorspheres was manually counted in each well per treatment group. DMSO vehicle concentration was equalized between all treatment groups in all experiments to control for any baseline vehicle toxicity.

### RNA-sequencing (RNA-Seq) workflow

U251MG cell cultures were drug treated for 48 h in 6-well tissue culture treated plates, trypsinized (0.05% trypsin–EDTA), pelleted, and RNA extracted at the University of Virginia Biorepository and Tissue Repository Facility using the RNeasy Lipid Tissue Mini Kit (cell pellet samples were stored at – 80 °C prior to RNA extraction), followed by RNA-Seq analysis at the UVA Genome Analysis and Technology Core facility. This involved RNA quality control using Agilent TapeStation RNA Kit, library preparations using NEBNext Ultra II Directional RNA Library Prep Kit (Illumina) and library quality control using Agilent TapeStation D5000 HS kit, followed by sequencing using NextSeq NGS NSQ2K kit P2 100 cycle kit (Illumina).

### Bioinformatic analysis for differential gene expression and impacted pathways

We received more than 30 million 61 bases-long paired end reads for each of the replicates sufficient for gene level quantitation. Read quality was assessed using fastqc program and raw data quality report was generated using the MultiQC tool. We used the “splice aware” aligner ‘STAR’ aligner for mapping reads, with more than 95% of reads mapping to the human genome and transcriptome. Gene-based read counts were derived from the aligned reads and a count matrix generated as input for differential gene expression analysis with the DESeq2 package. Low-expressed genes were filtered out. After raw count normalization using negative binomial normalization, we generated PCA plots for unsupervised clustering of control and treatment samples. Differentially expressed genes (DEGs) were ranked based on log2fold change and FDR corrected p-values. Additional noise reduction was done with adaptive shrinkage estimators using “apeglm” argument in “lfcShrink” function. The MA plot (function plotMA in DESeq2) and Volcano plot representing significant upregulated and downregulated genes was generated with functions in DESeq2. The heat map was generated with pheatmap package in R. The clusterprofiler (R-package) and msigdb pathway reference database were used for the pathway analysis. The reference database for pathway enrichment analysis comprised Hallmark, C2, and C5 gene sets from the msigdb. For each pathway analysis, we produced a list of the top 10 significant pathways based on adjusted p-value. Additionally, GSEA-style plots were generated for both upregulated and downregulated pathways.

### Phosphoproteomic workflow and data analysis

U251MG cells in 6-well tissue culture treated plates were drug treated for 24, 48, or 120 h followed by lysis with cell extraction buffer (Life Technologies FNN0011) containing protease and phosphatase inhibitors (Sigma). Lysates were clarified via centrifugation at 20,000×*g* for 15 min at 4 °C and total protein quantified using the Bicinchoninic Acid (BCA) protein assay (ThermoFisher) and normalized to 0.2–0.4 µg/µL for each sample. Samples received Luminex analysis using Quantitative Milliplex Multiplex Kit (Millipore Sigma) on the MAGPIX instrument with panels for multi-pathway phosphoproteins and TGF-β isoform quantification. Normalized (mean-centered and variance-scaled) data were projected in two-dimensions via Principal Component Analysis (PCA) using the *stats* package in R (version 4.2.2), similarly to a prior study by our group [31]. Importantly, the two-principal component projection captured the preponderance of variance among all samples (> 95%), with all sample treatment groups clustering independently.

### Caspase 3/7 activity and cell death inhibitor experiments

U251MG cells drug-treated in 96-well tissue culture plates for 48 h with DMSO, simvastatin, irinotecan, or simvastatin plus irinotecan were analyzed for caspase activity using the Caspase-Glo 3/7 Assay (Promega). Similar conditions were also tested with Z-VAD-FMK (pan-caspase inhibitor), chloroquine (autophagy inhibitor), necrostatin-1 (necroptosis inhibitor), or ferrostatin-1 (ferroptosis inhibitor) for 5-day treatment before CyQuant and crystal violet assays.

### TGF-β luciferase reporter (SBE4-luc) assays

U251MG or SB28 cells were plated and treated with drugs for either 48 h (U251MG) or 72 h (U251MG and SB28). Cells were transfected with SBE4-luc plasmid and co-transfected with PRL-Renilla plasmid using Fugene6 kit (Promega) per manufacturer’s recommendations, followed by incubation for 48 h prior to lysis and luminescence measurement on a Promega GloMax 20/20 manual luminometer using the Dual-Luciferase Reporter assay system kit (Promega). Results were double-normalized by first dividing SBE4-luc raw signal by PRL raw signal (to normalize viability loss) and then again normalized to mean DMSO ratio to show relative TGF-β signaling activity.

### Smad3 overexpression experiments

U251MG cells were seeded in 24-well tissue culture plates and transfected with BFP or Smad3CA plasmids for 48 h with Fugene6 per manufacturer’s instructions. After transfection, medium was replaced/repleted and cells were treated with DMSO, simvastatin, irinotecan, or simvastatin + irinotecan. Viability was measured after 96-h drug treatment using CyQuant assay.

### Exogenous TGF-β1 experiments

U251MG cells were seeded in 96-well tissue culture plates and treated with DMSO, simvastatin, irinotecan, or simvastatin + irinotecan with/without exogenous TGF-β1 protein. Viability was measured with CyQuant after 5 days.

### Statistical analysis

Statistical analysis and data plotting were done using Prism GraphPad software. Principal component analysis and RNA-Seq analysis plots were generated using R. For experiments involving comparison between groups for statistical significance, ANOVA with multiple comparisons was utilized. Bar plots were generated based on mean ± SEM (ns p > 0.05, *p < 0.05, **p < 0.01, ***p < 0.001, ****p < 0.0001). For quantitative determination of synergy, the Coefficient of Drug Interaction (CDI) was calculated as previously described [32, 33]. CDI < 1 indicates synergy, CDI < 0.7 indicates strong synergy, CDI = 1 indicates additivity, and CDI > 1 indicates antagonism [33]. The Bliss Combination Index (CI) was also used to assess synergy. CI for measured drug effect A (E_A_) and measured drug effect B (E_B_) was calculated as $CI= \frac{{E}_{A}+ {E}_{B}-{E}_{A}{E}_{B}}{{E}_{AB}}$, where E_AB_ is the measured effect of the drug combination A + B (CI < 1 suggests synergy, CI = 1 suggests additivity, CI > 1 suggests antagonism) [34, 35]. “Drug effect” for viability experiments refers to fractional cell killing, which was calculated by subtracting fraction of viable cells from 1.0.

## Results

### Simvastatin and irinotecan are synergistic in vitro in diverse GBM cellular models

**Fig. 1:**
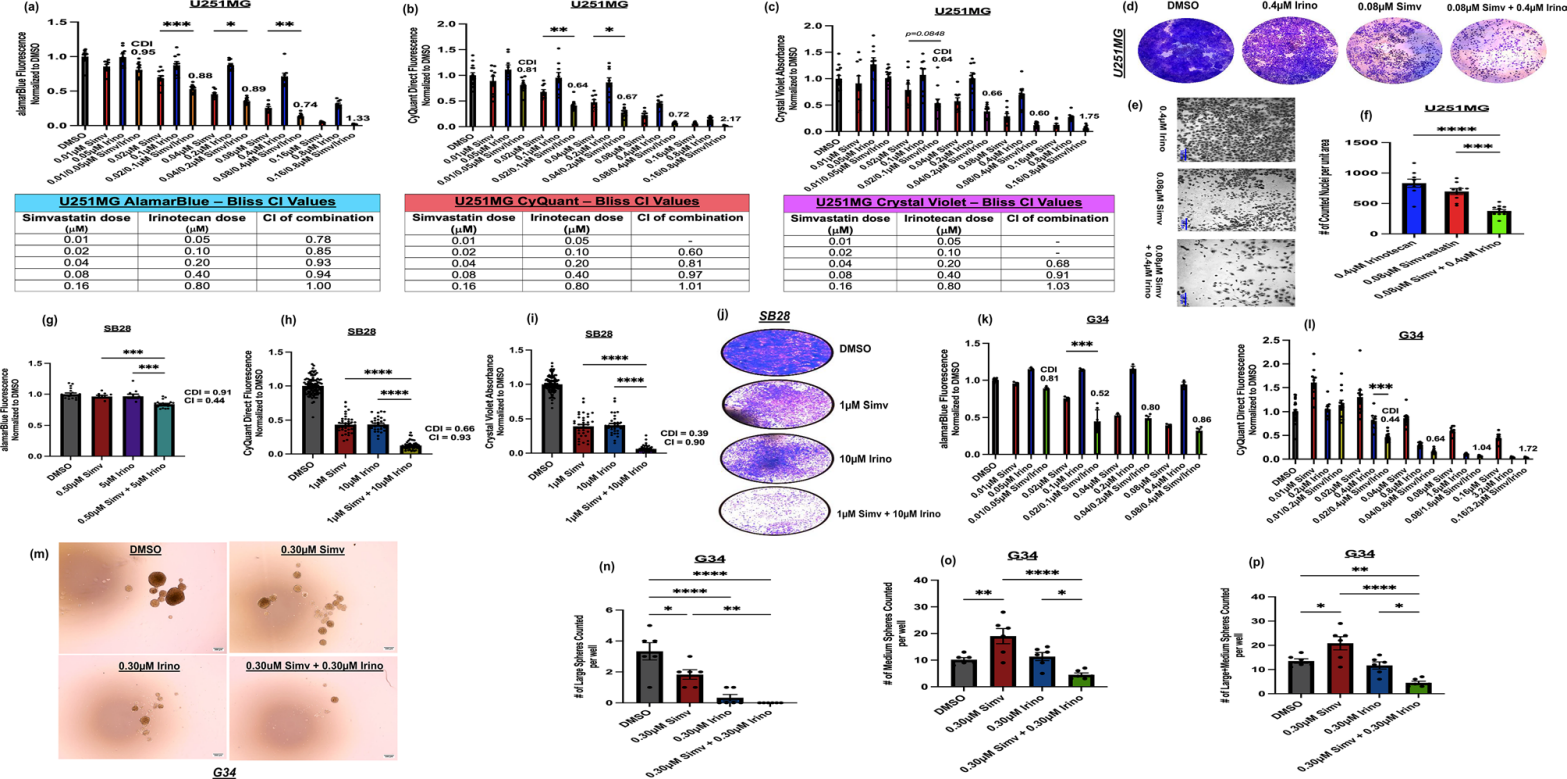
Simvastatin and irinotecan synergistically kill diverse GBM lines. *U251MG* human GBM line was treated for 5-days with DMSO (dimethyl sulfoxide vehicle control), simvastatin, irinotecan, or the combination at the indicated final concentrations and viability was then quantified with **a** AlamarBlue metabolic assay (fluorescence 544/590), **b** CyQuant Direct DNA proliferation assay (fluorescence 480/535), **c** Crystal violet staining assay quantification (absorbance OD570); **d** U251MG representative crystal violet whole-well images, **e**–**f** Brightfield microscopy images (scale bars 500 µm) and image segmentation nuclei quantification data. *SB28* murine GBM line was treated for 5-days with DMSO, simvastatin, irinotecan, or the combination at the indicated final concentrations and viability was then quantified with **g** AlamarBlue assay, **h** CyQuant assay, **i** Crystal violet staining assay, along with visualization of whole-well SB28 crystal violet staining (**j**). *G34* primary human glioma stem cells (GSCs) were treated for 5-days with DMSO, simvastatin, irinotecan, or the combination at the indicated final concentrations and viability was then quantified with **k** AlamarBlue assay, and **l** CyQuant assay. *G34* primary human GSCs were grown in serum-free, ultra-low attachment 3D culture conditions as primary tumorspheres and treated with either DMSO, simvastatin single-agent (simv), irinotecan single-agent (irino), or simvastatin + irinotecan combination. **m** Representative G34 tumorsphere images from each treatment group (scale bars 300 µm). Quantification of the number of **n** large, **o** medium, and **p** large + medium tumorspheres in each group. Large spheres were defined as at least ~ 300 µm in diameter with a dark core and medium spheres were defined as at least ~ 150 µm in diameter. Coefficient of drug interaction (CDI) values are indicated above each combination concentration ratio in panels (**a**–**c**), (**g**–**i**), (**k**, **l**) (CDI < 1 defined synergy) and Bliss CI values are indicated below each respective panel in table format for U251MG and within the plots for SB28 (CI < 1 defined synergy). Asterisks represent extent of statistical significance as explained under statistical analysis section of the text. All biological replicates (**n**) are depicted as distinct points in each respective panel, with numbers as follows: **a**–**c**, **f** n ≥ 9 per group, **g** n ≥ 10 per group, **h**, **i** n ≥ 33 per group, **k** n ≥ 3 per group, **l** n ≥ 9 per group, **n**–**p** n = 6 per group. No points were omitted from statistical analysis or from the graphs. Related data are shown in Supplementary Fig. 1

Mechanistically distinct cell viability assays based on cellular metabolism (alamarBlue), DNA proliferation (CyQuant), and cellular death/clearance (crystal violet staining) showed that simvastatin and irinotecan are synergistic at nanomolar doses in a cellular model of human GBM (U251MG). Specifically, alamarBlue assay (Fig. 1a) showed mathematical synergy across a nanomolar (less than 1 µM) dose range (CDI and CI less than 1) and the combination treatment showed significantly greater efficacy (p < 0.05) compared to the highest single-agent simvastatin activity at the 0.02 µM simvastatin/0.10 µM irinotecan, 0.04 µM simvastatin/0.20 µM irinotecan, and 0.08 µM simvastatin/0.40 µM irinotecan combinations. Concordance in trends was also seen with CyQuant assay (Fig. 1b), which showed synergy across the same concentration range (CDI and CI less than 1) and significantly greater activity than highest single-agent activity in the combination treatment (p < 0.05) for the 0.02 µM simvastatin/0.10 µM irinotecan and 0.04 µM simvastatin/0.20 µM irinotecan permutations. Similar concordance in trends was also seen with the crystal violet staining assay (Fig. 1c), which showed similar synergy across the same concentration range (CDI and CI less than 1) and significantly greater viability loss in the combination treatment at the 0.08 µM simvastatin/0.40 µM irinotecan condition both visually and quantitatively (Fig. 1d–f). Using a similar approach on the SB28 cellular model of murine GBM, we also observed quantitative synergy based on CDI and CI values (less than 1) and comparison of single agents to the combination (p < 0.05) (Figs. 1g–j, S1b). For the SB28 line, the 1 µM simvastatin/10 µM irinotecan permutation appeared to show the most dramatic synergy and concordance across multiple assays (Figs. 1h–j, S1b), though interestingly synergy using lower, less lethal concentrations (0.5 µM simvastatin and 5 µM irinotecan) was also seen in the alamarBlue assay (Fig. 1g). Synergy was similarly observed in primary glioma stem cells (GSCs) derived from patient neurosurgical samples (G34 line), at nanomolar (nM) concentrations. Using the alamarBlue assay, we noted the strongest synergy at the 0.02 µM simvastatin/0.10 µM irinotecan permutation (CDI = 0.52, p < 0.05 between simvastatin and combination treatment) (Fig. 1k). In the CyQuant assay, the strongest synergy was seen at the 0.02 µM simvastatin/0.40 µM irinotecan permutation (CDI = 0.44, p < 0.05 between irinotecan and combination treatment) (Fig. 1l). In all tested cases, however, synergy based on CDI < 1 was seen within a nanomolar-to-low-micromolar dose response across a dose range (Figs. 1k–l, S1a). Using primary tumorsphere formation assays on G34 GSCs, we also observed significant reduction in large and medium sphere formation by the various treatments at the tested concentrations (Fig. 1m–p). Simvastatin single-agent showed significant (p < 0.05) abrogation in large sphere formation (Fig. 1n) and significant increase in medium sphere formation (Fig. 1o) relative to DMSO control. Irinotecan single-agent also showed significant abrogation in large sphere formation (Fig. 1n), but did not show significant change in medium sphere formation (Fig. 1o), relative to DMSO control. The combination treatment, however, showed full ablation of large sphere formation relative to DMSO control (Fig. 1n), lowest medium sphere formation when compared to the single agent controls (Fig. 1o), and significantly lower large + medium sphere formation when compared to DMSO control (Fig. 1p), suggesting that the combination was the most effective treatment group in reducing GSC self-renewal at the tested conditions. Collectively, the results across the various tested GBM cellular models and assays support evidence of synergy in the combination treatment.

### RNA-Sequencing elucidates top genes and pathways engaged by simvastatin, irinotecan, and the combination treatment in human GBM

**Fig. 2:**
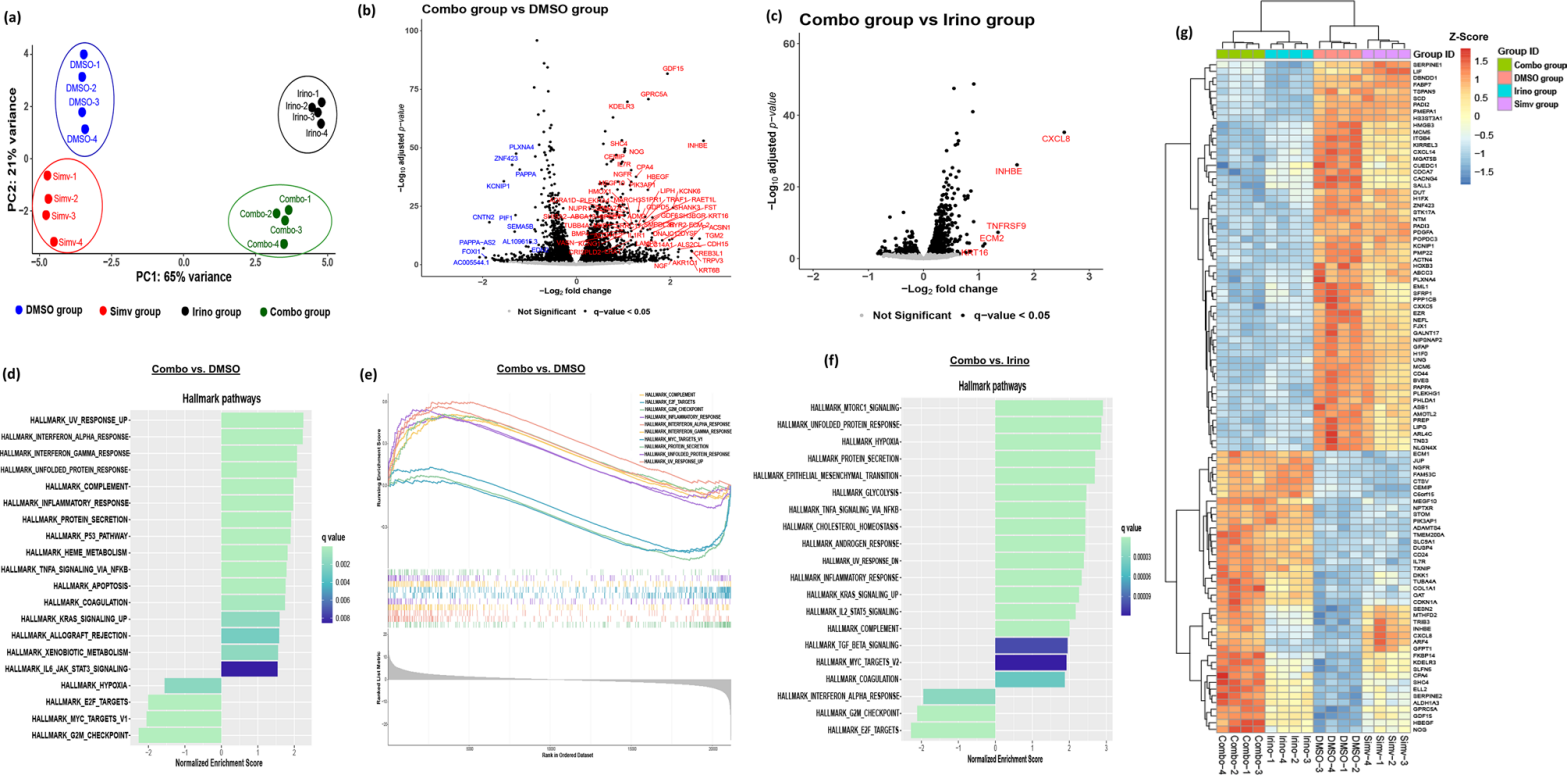
Bulk RNA-Seq elucidates top genes and pathways significantly dysregulated by treatment with the simvastatin + irinotecan combination. **a** Principal Component Analysis (PCA) of all treated groups (48 h treatment time) via unsupervised clustering (Simv = simvastatin, Irino = irinotecan, Combo = combination), **b** Volcano plot of differentially expressed genes 48 h after simvastatin/irinotecan combination treatment vs. DMSO control, **c** Volcano plot of differentially expressed genes 48 h after combination treatment vs. irinotecan single-agent, **d**, **e** Top molecular pathways enriched 48 h post-treatment in the combination treatment vs. DMSO, **f** Top molecular pathways enriched 48 h post-treatment in the combination treatment vs. irinotecan single-agent, **g** Heatmap of top 100 modulated genes 48 h post-treatment by simvastatin/irinotecan combination treatment vs. DMSO. Results are based on n = 4 biological replicates per treatment group (U251MG human GBM line). Cells were treated for 48 h with DMSO vehicle, 0.06 µM simvastatin single-agent, 0.60 µM irinotecan single-agent, or 0.06 µM simvastatin + 0.60 µM irinotecan (combination) prior to harvest. Heatmap of top 100 modulated genes in combination treatment vs. irinotecan single-agent contrast is shown in Supplementary Fig. 2

Using principal component analysis (PCA), we noted 86% of the variance in the data was captured across PC1 and PC2, with 65% on PC1 and 21% on PC2. Each of the four treatment conditions (DMSO, simvastatin single agent, irinotecan single agent, and simvastatin + irinotecan combination) clustered independently via unsupervised clustering (Fig. 2a). Volcano plot analysis revealed 4620 genes significantly dysregulated in the combination treatment vs. DMSO control based on criteria p_adj_ < 0.05, and 323 genes significantly modulated based on criteria (p_adj_ < 0.05, log_2_fold change > 1, log_2_fold change < − 1) (Fig. 2b). Based on PCA analysis, we noted that only 21% of the variance was explained between the combination vs. irinotecan groups (Fig. 2a), so we next sought to determine the genes significantly dysregulated between these groups that could be driving the observed effects in the combination treatment vs. irinotecan alone. Volcano plot analysis revealed 1172 genes significantly modulated in the combination vs. irinotecan contrast, based on criteria p_adj_ < 0.05 and five genes (*TNFRSF9, ECM2, INHBE, CXCL8, KRT16*) significantly modulated based on criteria (p_adj_ < 0.05, log_2_fold change > 1, log_2_fold change < − 1) (Fig. 2c). Pathway analysis elucidated significant upregulation of immunological pathways in the combination treatment vs. DMSO, specifically the interferon alpha, interferon gamma, TNF-α, complement and inflammatory gene pathways post-treatment in the combination group, as well as significant enrichment of stress/death pathways (unfolded protein response, DNA damage response, apoptosis), and oncogenic pathways (p53 pathway, KRAS signaling, JAK/STAT3 signaling, and hypoxia, among others) (Fig. 2d, e). When compared to irinotecan single-agent, the combination group interestingly showed significant enrichment of MTORC1 signaling, unfolded protein response, TNF-α signaling, cholesterol homeostasis, inflammatory response, KRAS signaling, and TGF-β signaling, among other pathways (Fig. 2f). Heatmap of the top 100 significantly modulated genes in combination vs. DMSO showed a subset synergistically upregulated, including *FKBP14, KDELR3, SLFN5, CPA4, SHC4, ELL2, SERPINE2, ALDH1A3, GPRC5A, GDF15, HBEGF*, and *NOG* (Fig. 2g). Notably, *SERPINE1*, a classic TGF-β target gene, was the top gene significantly affected by the drug combination vs. DMSO [7, 36–38] (Fig. 2g). In a similar manner, top gene hits were elucidated for the combination vs. irinotecan single-agent contrast (Supplementary Fig. 2).

### Targeted phosphoproteomics/proteomics implicates signaling pathways modulated by simvastatin, irinotecan, and the combination treatment in human GBM

**Fig. 3:**
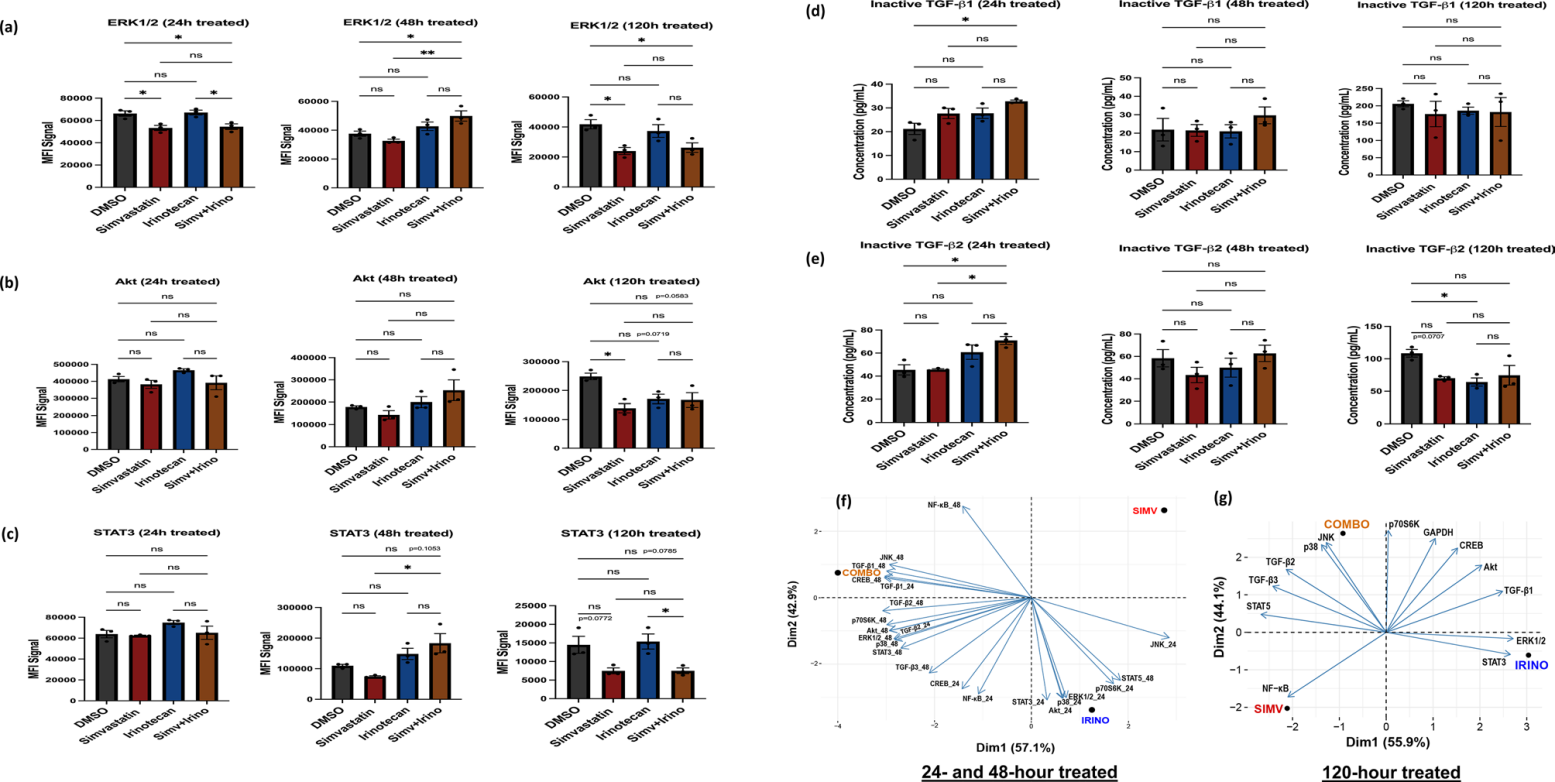
Targeted phosphoproteomics elucidates target hits at the translational/post-translational level with the simvastatin + irinotecan combination treatment. Raw mean fluorescence intensity (MFI) and analyte concentration plots of phosphoprotein molecular targets and biologically inactive TGF-β protein isoforms in the assayed panels at 24 h, 48 h, and 120 h post-treatment timepoints (TGF-β3 could not be plotted due to values below assay detection limit at certain timepoints) for **a** ERK 1/2, **b** Akt, **c** STAT3, **d** inactive TGF-β1, **e** inactive TGF-β2. Principal Component Analysis (PCA) biplots at **f** 24/48 h treatment timepoint and **g** 120 h treatment timepoint depicting target enrichment towards either simvastatin single-agent (SIMV), irinotecan single-agent (IRINO), or combination (COMBO; simv + irino) groups. Data are the result of n = 3 independent cell lysates per treatment group, with a unique set of lysates generated for each treatment timepoint from independent cell culture batches (U251MG human GBM line). Treatment concentrations for all timepoints were 0.04 µM simvastatin single-agent, 0.40 µM irinotecan single-agent, and 0.04 µM simvastatin + 0.40 µM irinotecan combination. Asterisks represent extent of statistical significance as explained under statistical analysis section of the text. Phosphoprotein targets with non-significant MFI trends across the entire time course are shown in Supplementary Fig. 3

Luminex analysis results showed significant changes in phosphoprotein levels of ERK/MAPK1/2, Akt/PKB, and STAT3, and protein levels of the TGF-β1 and TGF-β2 inactive isoforms in the combination treatment across the time course (Fig. 3a–e). Specifically, levels of phospho-ERK1/2 were significantly altered in the combination group at 24 h, 48 h, and 120 h post-treatment relative to DMSO (Fig. 3a). Levels of phospho-Akt were downregulated at 120 h post-treatment in combination vs. DMSO (Fig. 3b, p = 0.0583) and levels of phospho-STAT3 were significantly altered in combination treatment relative to simvastatin at 48 h post-treatment and significantly altered relative to irinotecan at 120 h post-treatment. Both TGF-β1 and TGF-β2 inactive protein isoforms showed significant upregulation in the combination treatment at 24 h post-treatment, relative to DMSO control (Fig. 3d, e). Interestingly, levels of TGF-β2 (but not TGF-β1) showed significant downregulation in irinotecan single-agent versus DMSO, at 120 h post-treatment (Fig. 3d, e). Phosphoprotein levels of CREB, JNK, NF-κB, p38, p70S6K, and STAT5 were not significantly altered at any of the tested timepoints (Supplementary Fig. 3a–f). These observations support involvement of TGF-β in synergistic activity of the combination, as well as potential roles for other signaling nodes. Notably, significant enrichment of KRAS signaling in transcriptomic analysis at 48 h post-treatment (Fig. 2d, f) and significantly elevated phosphoprotein levels of ERK 1/2 in the combination group at 48 h of treatment (Fig. 3a) suggest a role for the RAS/RAF/MEK/ERK signaling pathway in synergistic activity—or possibly up-regulation as a compensatory mechanism. Furthermore, most protein targets in the assayed panels projected towards the combination treatment group, especially at the 24- and 48-h timepoints, with 100% of sample variance captured across both principal component dimension 1 (Dim1) and principal component dimension 2 (Dim2) (Fig. 3f, g).

### Simvastatin and irinotecan synergistically increase caspase 3/7 activity (apoptosis) and engage additional death pathways in human GBM cells

**Fig. 4:**
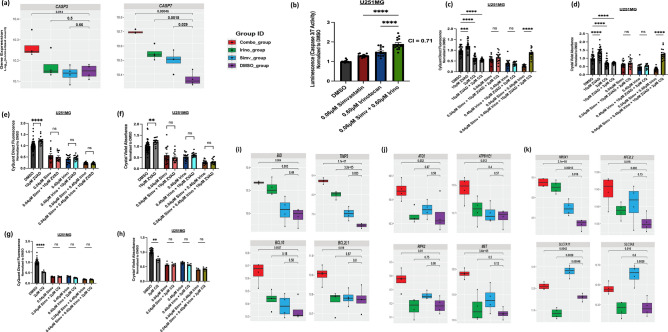
Simvastatin and irinotecan combination treatment simultaneously engages multiple cell death pathways. **a** Bulk RNA-Seq boxplots depicting *caspase3* and *caspase7* gene expression in DMSO vs. simvastatin single-agent (Simv) vs. irinotecan single-agent (Irino) vs. simvastatin + irinotecan combination (Combo) treated groups at 48 h post-treatment, **b** Caspase-Glo 3/7 activity assay after 48 h of treatment with DMSO, simvastatin, irinotecan, or the combination at the indicated concentrations shows increased activity with the individual drugs and combination, **c** CyQuant Direct DNA proliferation assay and **d** Crystal violet absorbance quantification after 5-days of treatment with DMSO, simvastatin, irinotecan, or the simvastatin + irinotecan combination with and without a pan-caspase inhibitor (Z-VAD-FMK) and autophagy inhibitor (chloroquine) present. **e**–**f** CyQuant and crystal violet viability quantification with simvastatin/irinotecan with/without only Z-VAD-FMK inhibitor co-treatment, **g**, **h** CyQuant and crystal violet viability quantification with simvastatin/irinotecan with/without only chloroquine inhibitor co-treatment **i** Bulk RNA-Seq gene boxplots depicting significant upregulation of representative programmed cell death (primarily apoptosis)-associated genes (*BID, TIMP3, BCL10, BCL2L1)* with the combination treatment*.* **j** Bulk RNA-Seq gene boxplots depicting significant upregulation of representative autophagy associated genes (*ATG5, ATP6V1E1, RIPK2, MET)* with the combination treatment*.* **k** Bulk RNA-Seq gene boxplots depicting significant upregulation of representative ferroptosis associated genes (*HMOX1, NFE2L2, SLC7A11, SLC3A2).* All data are at least n = 4 biological replicates per group (U251MG human GBM line). Related data are shown in Supplementary Fig. 4. Asterisks represent extent of statistical significance as explained under statistical analysis section of the text. Bliss combination index (CI) is shown above the combination group in (**b**) [CI < 1 indicates synergy]. Adjusted p-values in each treatment condition compared to DMSO are shown within the gene expression boxplots in (**a**), (**i**–**k**)

Transcriptomic analysis showed the greatest increase in expression of key apoptotic genes *CASP3* and *CASP7* in the combination treatment compared to single agents, based on p-value comparison to DMSO (Fig. 4a). Experimental validation with a caspase 3/7 activity assay confirmed a synergistic increase in caspase 3/7 activity with the combination relative to the individual agents at the same concentrations (Bliss CI < 1), implicating synergistic activation of the apoptosis pathway (Fig. 4b). Interestingly, however, a pan-caspase inhibitor (Z-VAD-FMK) failed to rescue viability loss from the combination treatment when used individually, suggesting compensatory caspase-independent death pathways with combination treatment (Fig. 4e, f) or potential efflux of Z-VAD-FMK from GBM cells [39]. The former was supported by transcriptomic data, which showed significant upregulation of genes related to autophagy with the combination treatment (*ATG5, ATP6V1E1, RIPK2, MET,* among others [40–42]) (Figs. 4j, S4e). Interestingly, however, an autophagy inhibitor (chloroquine) also failed to rescue viability loss from the combination treatment when used individually (Fig. 4g, h). Results also showed significant upregulation of genes related to ferroptosis (*HMOX1, NFE2L2, SLC7A11, SLC3A2 *[43–45]) (Figs. 4k, S4f), in addition to significant upregulation of genes associated with various programmed death pathways (*BID, TIMP3, BCL10, BCL2L1,* among others [46–48]) (Figs. 4i, S4a–d). We attempted to individually inhibit these pathways with relevant pharmacological inhibitors (necrostatin-1 for necroptosis and ferrostatin-1 for ferroptosis), but did not see evidence for significant viability rescue (Supp Fig. 4g–j). However, when we combined a pan-caspase inhibitor (Z-VAD-FMK) with an autophagy inhibitor (chloroquine), we noted significant rescue of viability in the combination treatment (Fig. 4c, d), confirming the importance of both caspase-dependent pathways and autophagy together in the killing mechanism of the combination treatment.

### Functional assays corroborate gene expression data to suggest TGF-β signaling as a mechanism for GBM cell killing by irinotecan and irinotecan + simvastatin in GBM

**Fig. 5:**
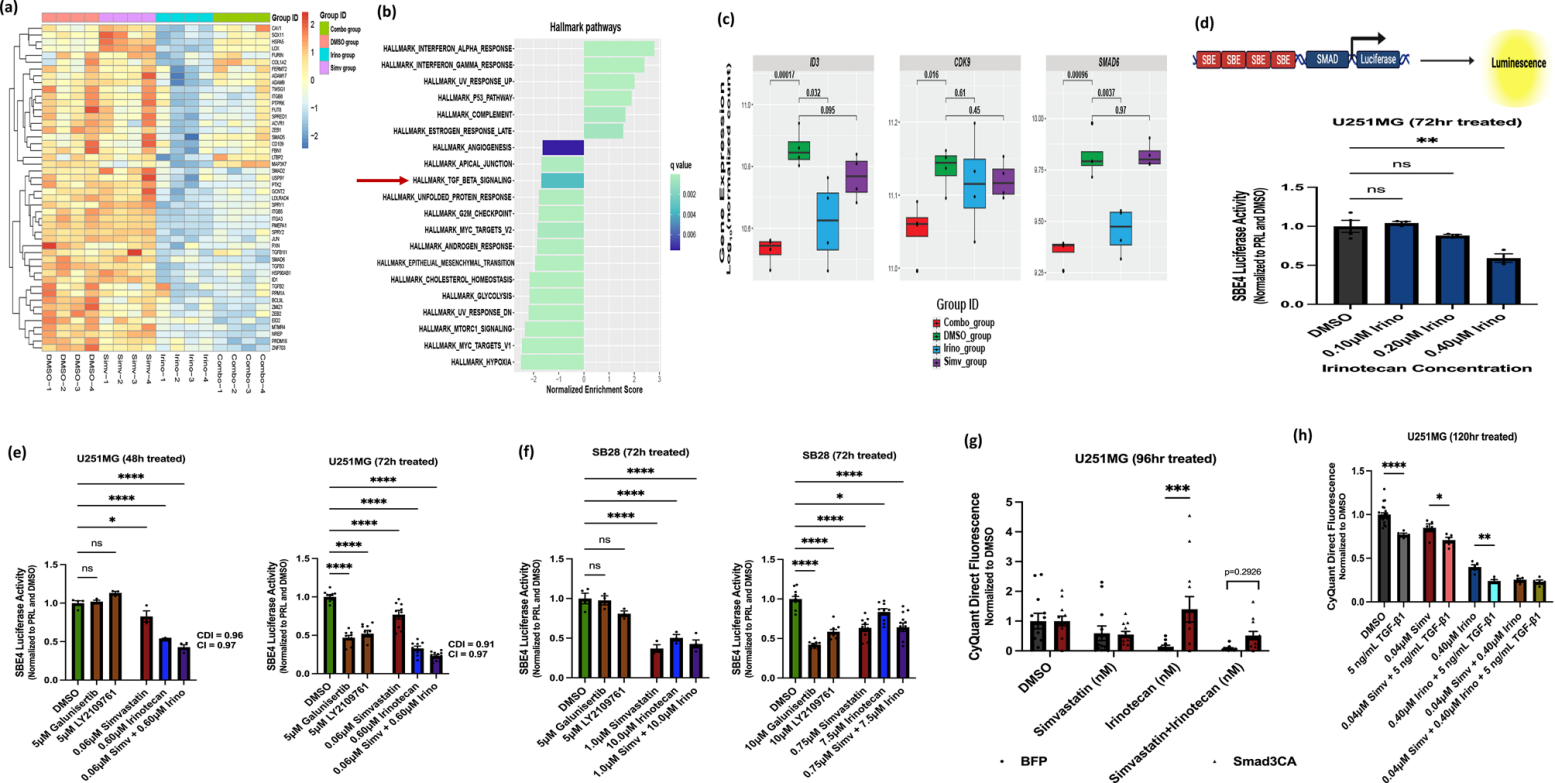
Irinotecan and irinotecan + simvastatin treatments converge on TGF-β signaling as a mechanism for GBM cell killing. **a** RNA-Seq gene expression heatmap showing differentially expressed genes related to TGF-β receptor signaling pathway (Irinotecan vs. DMSO contrast). **b** RNA-Seq of top molecular pathways significantly enriched by irinotecan treatment (vs. DMSO), with TGF-β signaling downregulation marked with a red arrow. **c** RNA-Seq gene expression boxplots depicting significant downregulation of representative TGF-β–associated genes with the combination (combo) treatment (*ID3, CDK9, SMAD6)*. **d** *Top:* Schematic of SBE4-luciferase reporter construct to model canonical TGF-β signaling activity; *Bottom:* Irinotecan single-agent dose-dependently decreases TGF-β signaling reporter activity. **e** Irinotecan and simvastatin significantly decrease TGF-β signaling reporter activity (alone and in combination) relative to commercial TGF-β signaling inhibitors (galunisertib, LY2109761) in U251MG human GBM line. **f** Irinotecan and simvastatin significantly decrease TGF-β signaling reporter activity (alone and in combination) relative to commercial TGF-β signaling inhibitors in SB28 murine GBM line. **g** Overexpression of constitutively active SMAD3 (downstream TGF-β signaling mediator) in GBM cells via transient transfection differentially rescues viability loss from irinotecan single-agent treatment. **h** Exogenous TGF-β1 protein sensitizes viability loss to simvastatin and irinotecan single agents. All data is mean ± SEM of at least n = 3 biological replicates. Cell line and treatment time information is depicted above plots. Asterisks represent extent of statistical significance as explained under statistical analysis section of the text. Adjusted p-values in each treatment condition compared to DMSO are shown within the gene expression boxplots in (**c**). Related data are shown in Supplementary Fig. 5

Through transcriptomic analysis, we noted that irinotecan alone and in the combination treatment significantly decreases expression of key TGF-β pathway genes (Figs. 5a-c, S5) [7, 49–51]. Using the SBE4-luciferase reporter [7], which contains 4 SMAD-binding elements upstream of a constitutive promoter driving firefly luciferase (Fig. 5d), we noted significant reduction of canonical TGF-β signaling activity with simvastatin and irinotecan, both individually and in combination, in both U251MG and SB28 lines (Fig. 5d–f). Importantly, these agents inhibited TGF-β signaling comparably or more so than commercially available inhibitors of canonical TGF-β signaling (Fig. 5e, f). Following transient overexpression of constitutively-active SMAD3, a key mediator of TGF-β signaling, we noted significant rescue of GBM cytotoxicity from irinotecan and partial rescue of irinotecan + simvastatin cytotoxicity (Fig. 5g). Interestingly, adding exogenous TGF-β1 yielded significant sensitization to both simvastatin and irinotecan individually, but not the combination (Fig. 5h). The findings with simvastatin monotherapy are consistent with our prior report [7].

## Discussion

These results demonstrate in vitro synergistic efficacy of simvastatin and irinotecan in three diverse GBM cellular models, including U251MG (well-established workhorse model for human GBM originally derived from a GBM patient), G34 (primary, patient-derived GSC model from neurosurgical specimens), and SB28 (genetically-derived murine GBM model), whose genetic and phenotypic characteristics as GBM models have been recently reviewed and characterized [29, 30, 52]. Importantly, in both models of human GBM (U251MG and G34), we observed synergistic anti-GBM activity combining simvastatin and irinotecan at nanomolar concentrations (i.e. less than 1 µM), which align with concentrations achievable in patients [18, 53]. Specifically, simvastatin has been previously reported to achieve mean and maximum concentrations in human serum at high nanomolar levels [53–55] and has been reported to have modest blood–brain barrier (BBB) permeability given its lipophilicity [8–11]. A prior report on clinically relevant concentrations of anti-cancer drugs has noted that irinotecan in its baseline form achieves maximum plasma concentration (C_max_) of approximately 6 µM in patients and approximately 63 µM in its liposomal form; its active component, SN-38, has been reported to achieve C_max_ up to 0.143 µM [56]. Additionally, irinotecan has been reported to also have modest BBB permeability [18, 19, 57]. In view of these pharmacokinetic data, we posit that the present study is clinically and translationally relevant.

This combinatorial GBM killing by simvastatin and irinotecan correlated with increased elevation of caspase 3/7 expression and activity at the transcriptomic and protein levels, implicating a role for apoptosis in mediating synergy. Additionally, roles for other pathways were suggested by transcriptomic analysis, which showed significantly upregulated expression of genes associated with autophagy and ferroptosis in the combination therapy. Interestingly, combining an autophagy inhibitor (chloroquine) and a caspase inhibitor (Z-VAD-FMK) with the simvastatin + irinotecan combination significantly rescued/abrogated viability loss, suggesting that *both* apoptosis and autophagy are important for GBM viability loss with the combination.

Through transcriptomic analysis, we noted significant downregulation of many genes in the TGF-β family, including its classic target gene, *SERPINE1* [7, 36–38]*,* in both the irinotecan and irinotecan + simvastatin treatment groups; we hypothesize this is likely due to the transcriptional inhibition activities of topoisomerase I inhibitors [58]. Functional studies validated significant inhibition of TGF-β signaling in multiple GBM lines (U251MG, SB28), which is significant given its pro-tumorigenic role in GBM and other cancers via both tumor-intrinsic and extrinsic (i.e. immune microenvironment) mechanisms [20–22, 24–27]. Importantly, for irinotecan and irinotecan + simvastatin, we noted comparable or greater TGF-β inhibitory activity compared to commercial inhibitors of the same pathway, at equal or lower concentrations, thus highlighting the potential of these agents as clinically relevant TGF-β inhibitors—which is especially pertinent given the lack of dedicated TGF-β inhibitors in the clinic. Notably, irinotecan inhibition of TGF-β signaling has not been previously reported. Additional studies involving exogeneous addition of an upstream activator (TGF-β1 protein) demonstrated significant sensitization of GBM cells to irinotecan and simvastatin, potentially indicating that TGF-β up-regulation may be “addicting” GBM to this pathway and sensitizing to TGF-β inhibition by irinotecan plus simvastatin. In contrast, the constitutively-active Smad3 significantly rescues GBM cells from irinotecan single-agent cytotoxicity (and partially rescues from combination treatment toxicity), suggesting that irinotecan toxicity is partially mediated by TGF-β inhibition and that adding simvastatin alters how TGF-β is inhibited. Taken together, our findings of (1) significant downregulation of the *SERPINE1* gene (a key TGF-β target gene in cancer) with the combination treatment (Fig. 2g), (2) significant downregulation of gene expression of many TGF-β family members by irinotecan and the combination (Figs. 5a–c, S5), and (3) consideration of our prior 2019 report demonstrating that simvastatin toxicity against GBM is mediated by TGF-β [7], in conjunction with (4) systematic interrogation of the engagement of TGF-β pathway using genetic/biochemical/functional validation assays (Fig. 5d–h), show convergence of GBM killing mechanism on the TGF-β signaling pathway with the combination (simvastatin + irinotecan) treatment.

In addition to TGF-β, we also noted significant modulation of ERK/MAPK1/2 phosphoprotein levels, which was correlated with significant enrichment of the KRAS signaling pathway in transcriptomic analysis; this may be significant given the role of RAS signaling in oncology and GBM [59]. Moreover, the transcriptomic data also showed significant enrichment of pathways associated with immune responses, including interferon alpha, interferon gamma, complement, inflammatory response, and TNF-α signaling, thus highlighting potential immunotherapy potential of this drug combination via immunogenic cell death of GBM cells [60]—which typically has an immunologically “cold” microenvironment [61].

## Conclusions and limitations

Collectively, these results demonstrate promising efficacy of a novel combination treatment utilizing the repurposed drug simvastatin with irinotecan chemotherapy against GBM at clinically feasible concentrations. This is the first study to delineate the underlying molecular targets and pathways affected by the simvastatin/irinotecan treatment in diverse GBM lines using global and targeted approaches. Moreover, this is the first demonstration that irinotecan inhibits TGF-β in GBM, and that the simvastatin + irinotecan combination converges on this pathway—offering a potential approach to TGF-β inhibition in the clinic. However, it should be noted that the present study did not include in vivo assessment of survival or tumor volume with the combination treatment in animal models of intracranial GBM, which represents a key limitation. Current efforts are underway by our group to assess potential in vivo efficacy of the combination treatment against GBM.

## Supplementary Information

Below is the link to the electronic supplementary material.

Fig. S1: Simvastatin and irinotecan combination treatment decreases GBM viability in a dose-dependent manner. (a) G34 viability assessment with alamarBlue assay at same concentrations as CyQuant assay shown in Figure 1, (b) SB28 viability assessment with alamarBlue assay at same concentrations as CyQuant and Crystal Violet assay shown in Fig. 1. Coefficient of drug interaction (CDI) and/or Bliss Combination Index (CI) values are indicated above each combination concentration ratio. Asterisks represent extent of statistical significance as explained under statistical analysis section of the text. All biological replicates (n) are depicted as distinct points in each respective panel, with numbers as follows: (a) n ≥ 9 per group, (b) n ≥ 10 per group. No points were omitted from statistical analysis or from the graphs. Supplementary file1 (PDF 370 KB)

Fig. S2: Top differentially expressed genes between combination and irinotecan treatment groups. Gene heatmap showing top 100 differentially expressed genes in combination treatment vs. irinotecan single-agent contrast. All data are n = 4 biological replicates per group (U251MG human GBM line). Supplementary file2 (PDF 285 KB)

Fig. S3: Simvastatin + irinotecan combination treatment does not significantly modulate certain selected phosphoprotein targets. Raw mean fluorescence intensity (MFI) of phosphoprotein targets in the assayed panels at 24 h, 48 h, and 120 h post-treatment timepoints for (a) CREB, (b) JNK/SAPK1, (c) NF-κB, (d) p38/SAPK 2A/B, (e) P706K, (f) STAT5A/B. Data are the result of n = 3 independent cell lysates per treatment group, with a unique set of lysates generated for each treatment timepoint from independent cell culture batches (U251MG human GBM line). Supplementary file3 (PDF 1967 KB)

Fig. S4: Simvastatin + irinotecan combination treatment upregulates gene signatures associated with multiple cell death pathways. Gene expression heatmaps showing differentially expressed genes related to (a) Apoptosis pathway, (b) Intrinsic apoptotic signaling pathway, (c) Extrinsic apoptotic signaling pathway, (d) Apoptotic cell clearance pathway, (e) Autophagy pathway, (f) Ferroptosis pathway. (g) CyQuant and (h) Crystal violet quantification of viability loss with simvastatin, irinotecan, simvastatin + irinotecan with/without 10 uM necrostatin1 (necroptosis inhibitor) after 5 days of treatment; (i) CyQuant and (j) Crystal violet quantification of viability loss with simvastatin, irinotecan, simvastatin + irinotecan with/without 10 uM ferrostatin1 (ferroptosis inhibitor) after 5 days of treatment. Data are the result of n ≥ 4 biological replicates per treatment group (U251MG human GBM line). Supplementary file4 (PDF 1146 KB)

Fig. S5: Irinotecan alone and in combination with simvastatin decreases gene expression of TGF-β pathway family members. Bulk RNA-Seq boxplots depicting gene expression of selected TGF-β pathway family members (*SERPINE1, RHOA, TGFβR1) *in DMSO vs. simvastatin single-agent (Simv) vs. irinotecan single-agent (Irino) vs. simvastatin + irinotecan combination (Combo) treated groups at 48 h post-treatment. All data are n = 4 biological replicates per group (U251MG human GBM line). Adjusted p-values in each treatment condition compared to DMSO are shown within the gene expression boxplots. Supplementary file5 (PDF 34 KB)

## Data Availability

RNA-Sequencing data generated in this study have been deposited into a publicly available database for access.
