# Supplementary figures and images for: Synergistic activity of simvastatin and irinotecan chemotherapy against glioblastoma converges on TGF-β signaling
Source: J Neurooncol. 2025 May 28;174(3):621–33. doi: 10.1007/s11060-025-05089-8 (PMC12263772; doi:10.1007/s11060-025-05089-8)

(a)

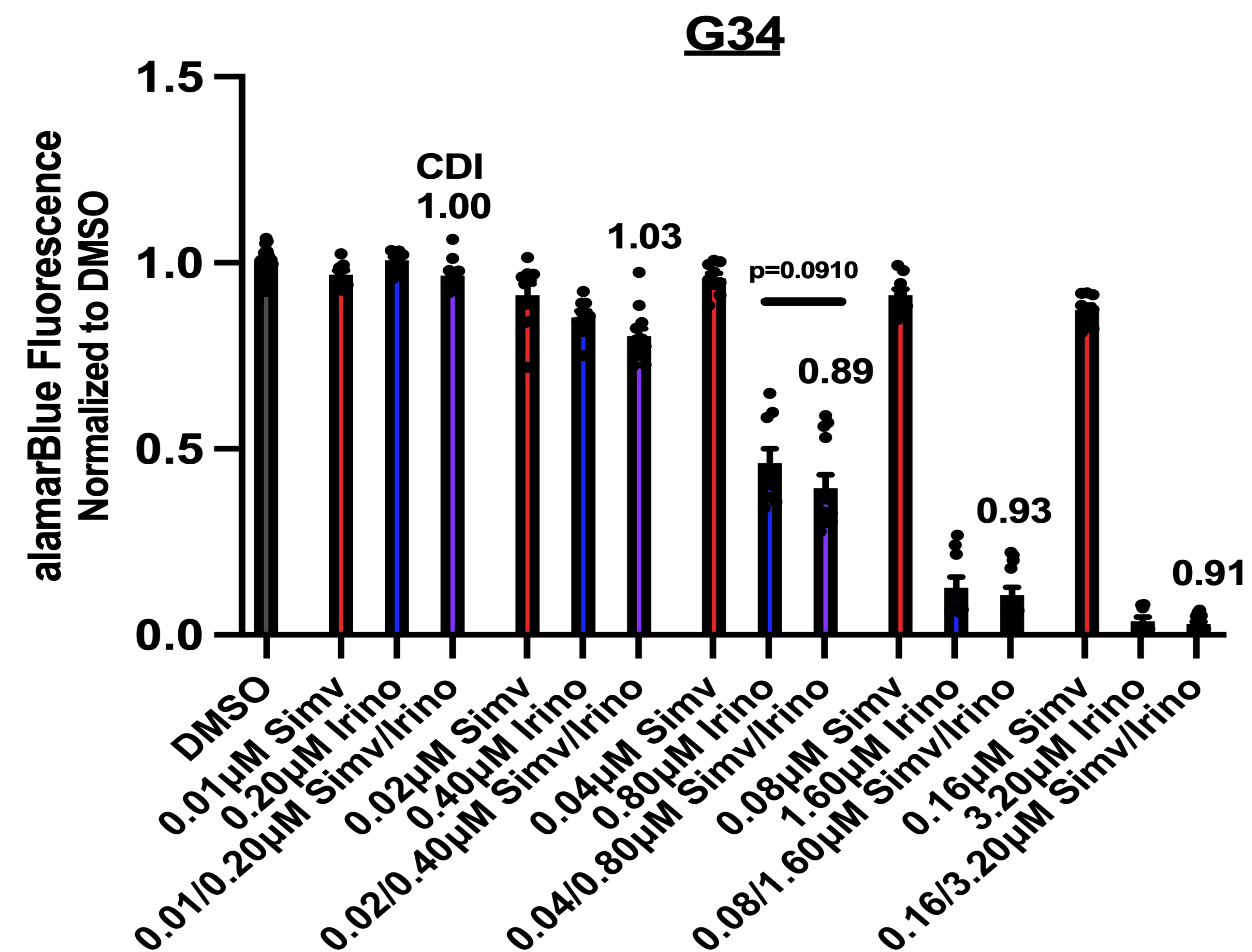

(b)

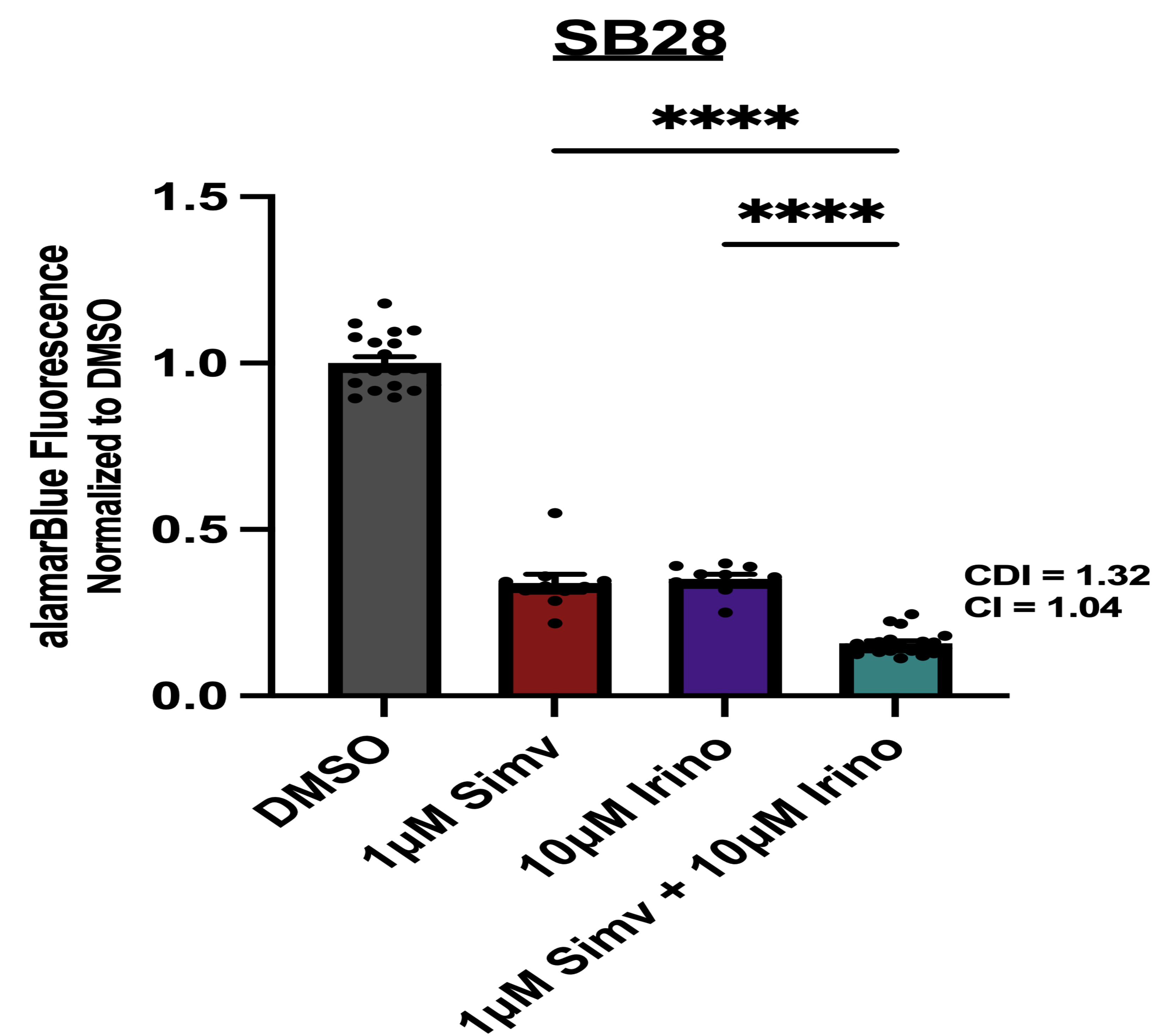

Supplement: Supplementary file 1 — Fig. S1: Simvastatin and irinotecan combination treatment decreases GBM viability in a dose-dependent manner. (a) G34 viability assessment with alamarBlue assay at same concentrations as CyQuant assay shown in Figure 1, (b) SB28 viability assessment with alamarBlue assay at same concentrations as CyQuant and Crystal Violet assay shown in Fig. 1. Coefficient of drug interaction (CDI) and/or Bliss Combination Index (CI) values are indicated above each combination concentration ratio. Asterisks represent extent of statistical significance as explained under statistical analysis section of the text. All biological replicates (n) are depicted as distinct points in each respective panel, with numbers as follows: (a) n ≥ 9 per group, (b) n ≥ 10 per group. No points were omitted from statistical analysis or from the graphs. Supplementary file1 (PDF 370 KB) [file 11060_2025_5089_MOESM1_ESM.pdf]

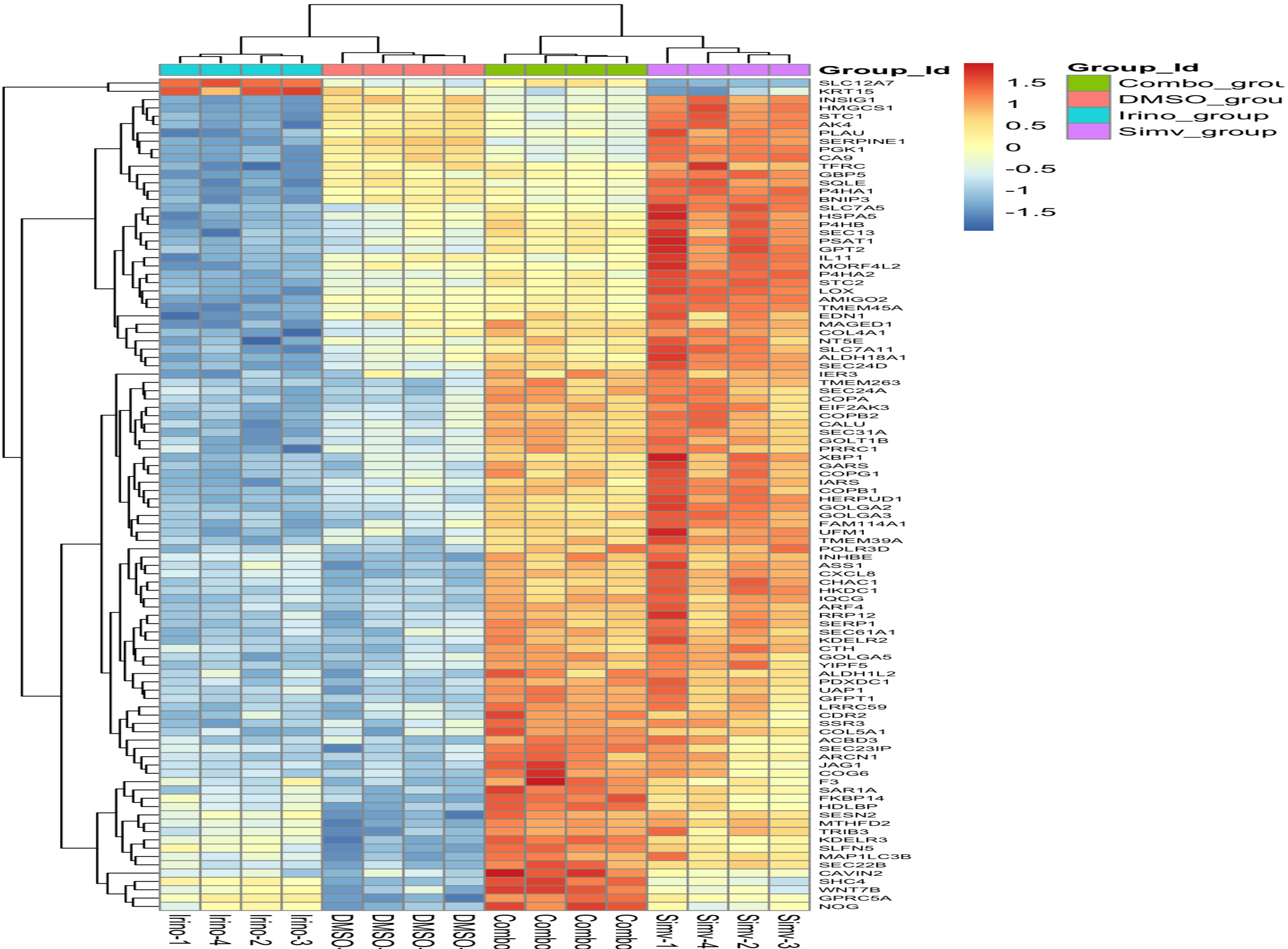

Supplement: Supplementary file 2 — Fig. S2: Top differentially expressed genes between combination and irinotecan treatment groups. Gene heatmap showing top 100 differentially expressed genes in combination treatment vs. irinotecan single-agent contrast. All data are n = 4 biological replicates per group (U251MG human GBM line). Supplementary file2 (PDF 285 KB) [file 11060_2025_5089_MOESM2_ESM.pdf]

## CREB

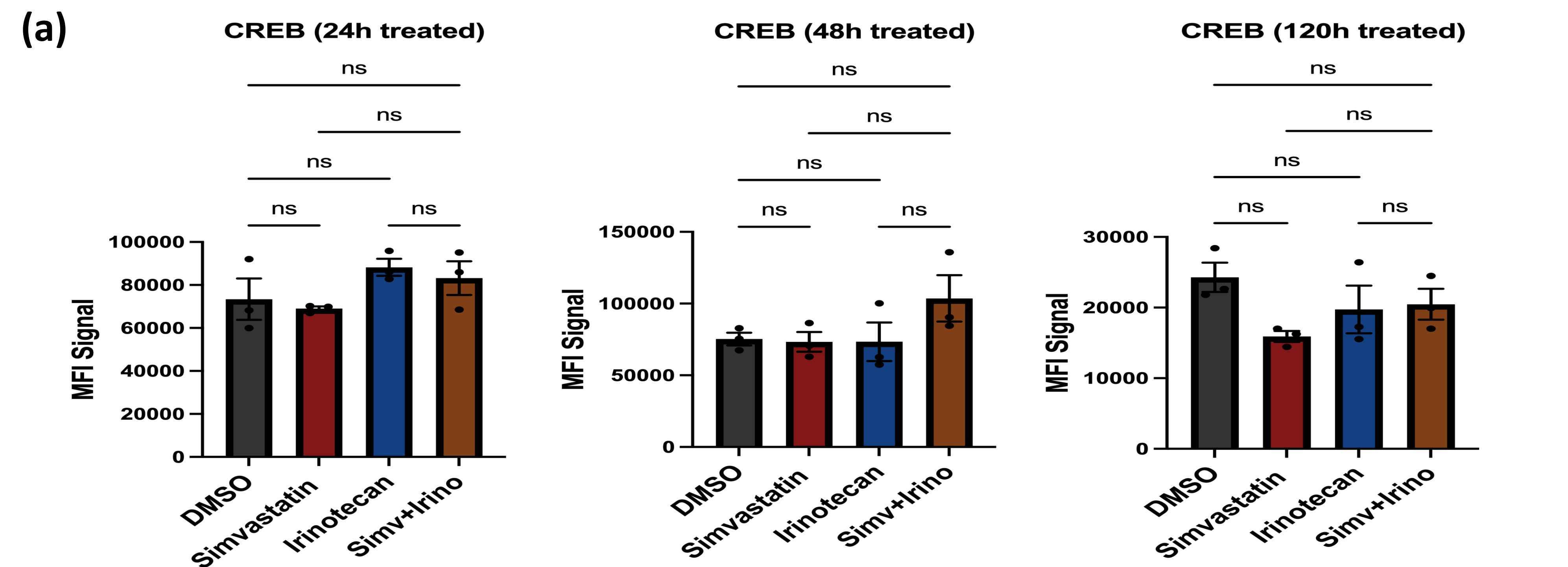

## JNK/SAPK1

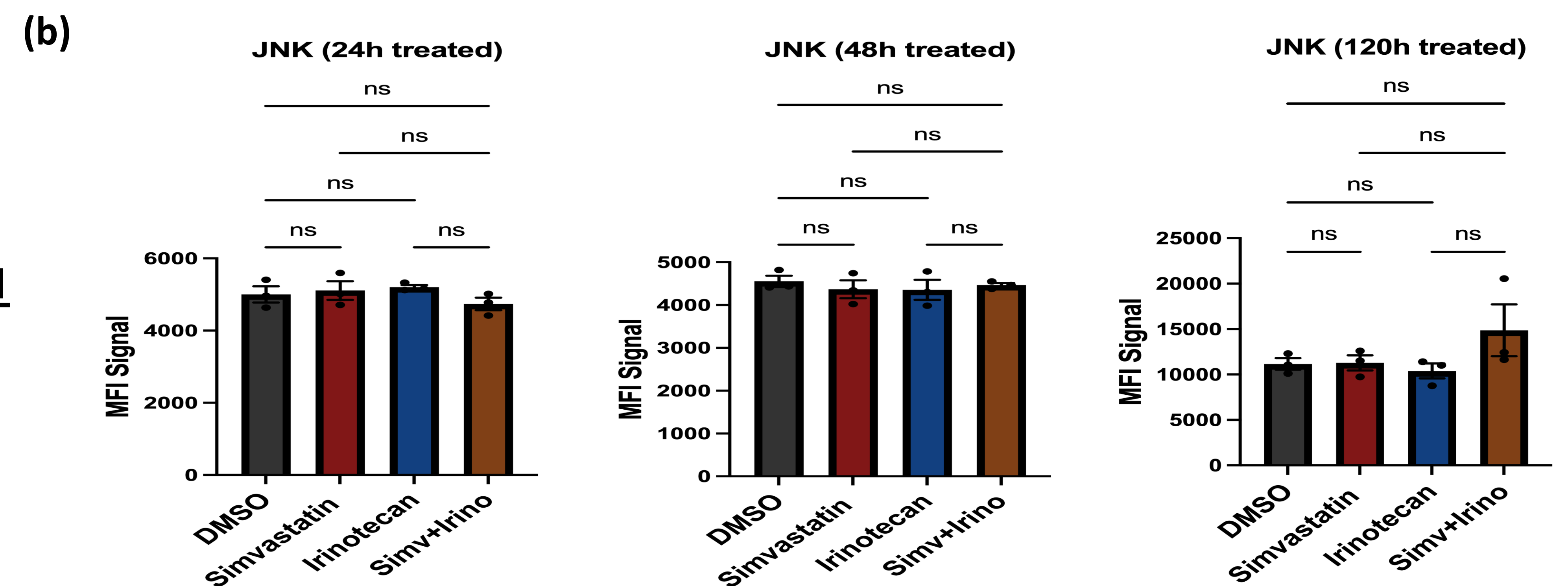

**NF-κB**

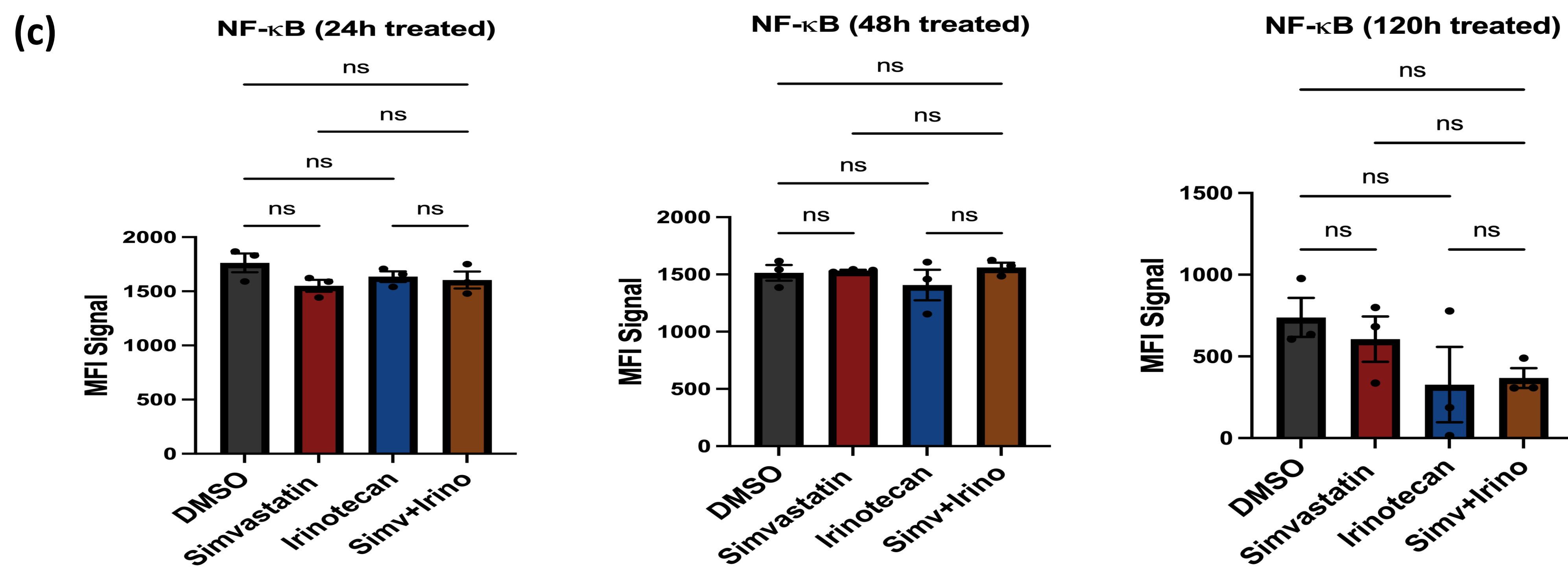

**p38/SAPK2A/B**

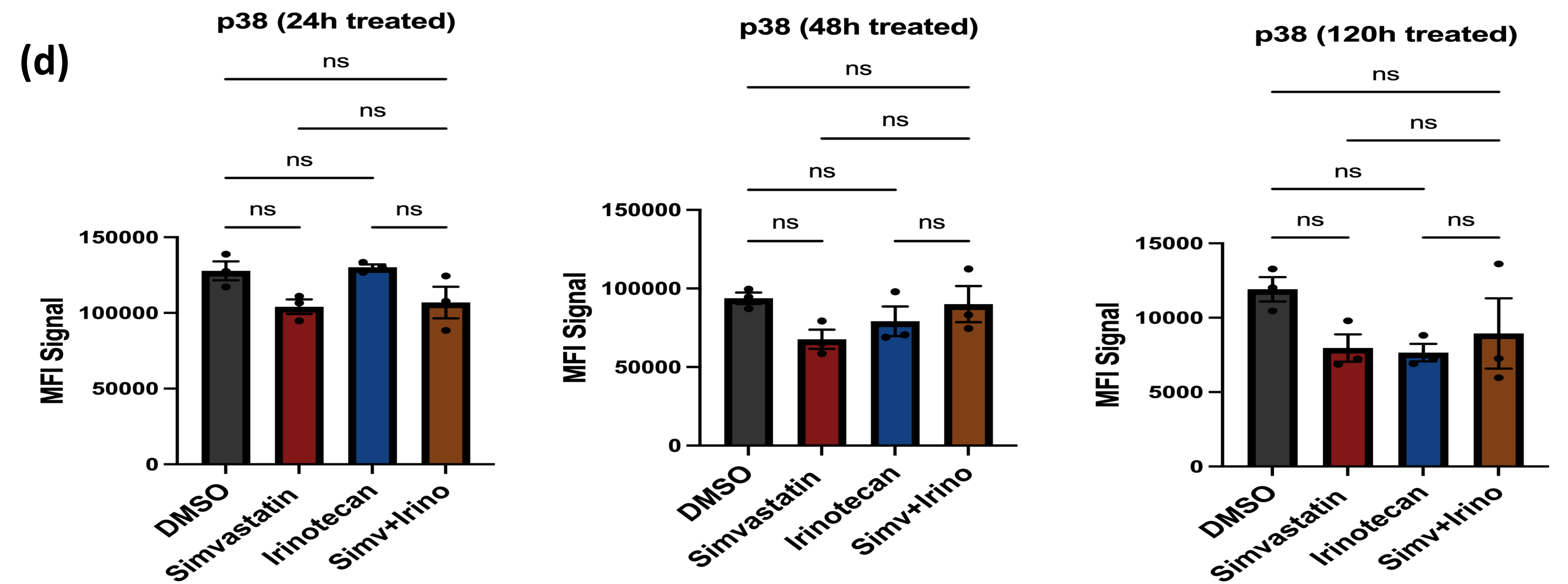

**P706K**

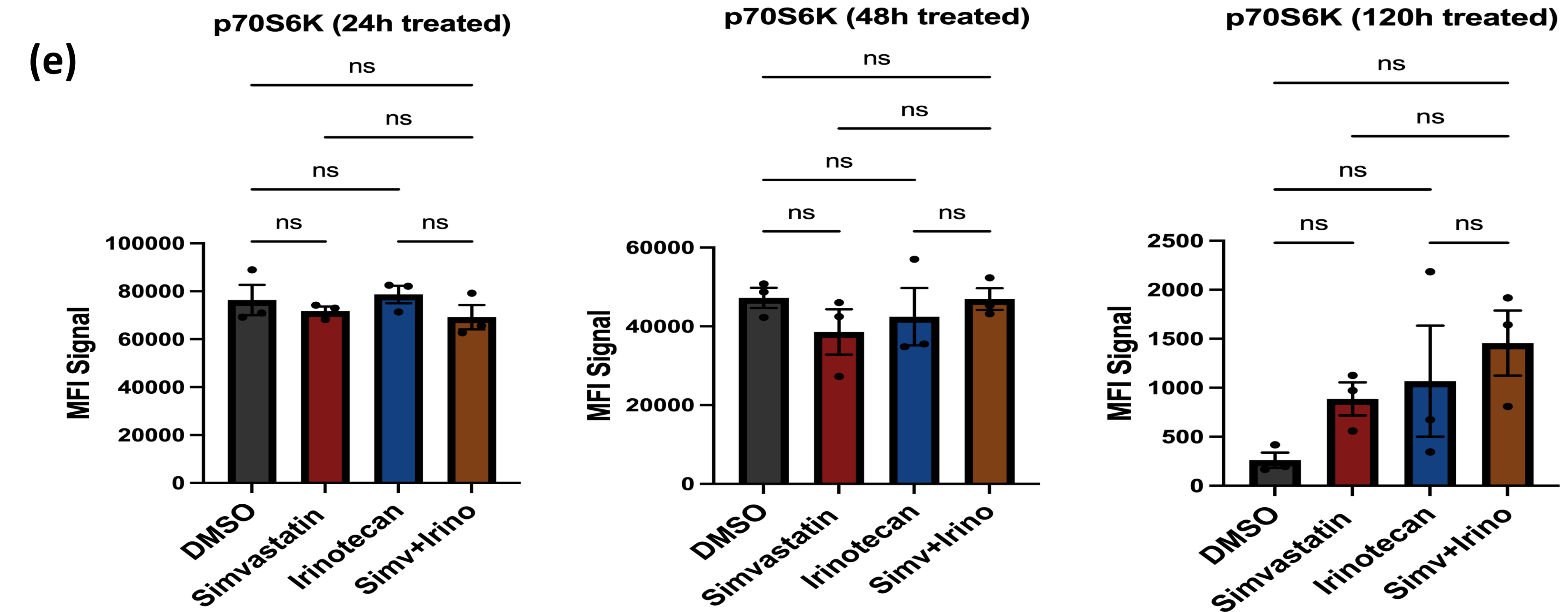

**STAT5A/B**

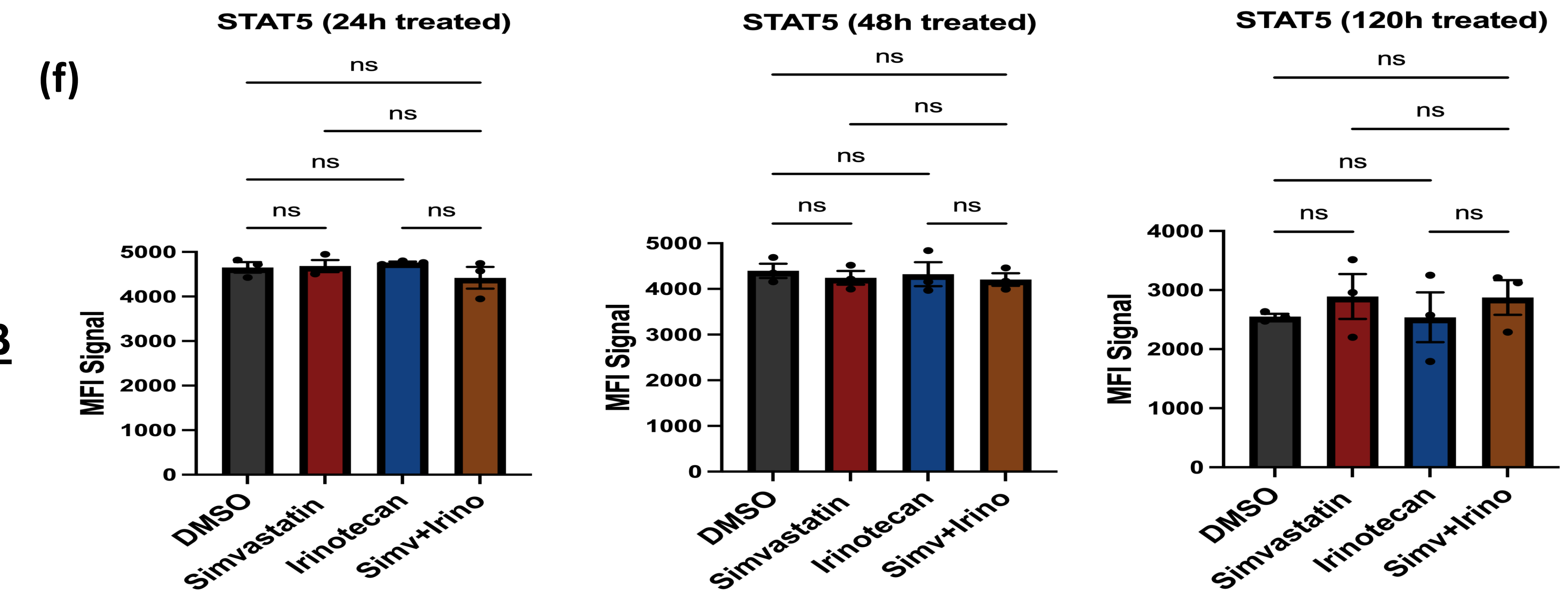

Supplement: Supplementary file 3 — Fig. S3: Simvastatin + irinotecan combination treatment does not significantly modulate certain selected phosphoprotein targets. Raw mean fluorescence intensity (MFI) of phosphoprotein targets in the assayed panels at 24 h, 48 h, and 120 h post-treatment timepoints for (a) CREB, (b) JNK/SAPK1, (c) NF-κB, (d) p38/SAPK 2A/B, (e) P706K, (f) STAT5A/B. Data are the result of n = 3 independent cell lysates per treatment group, with a unique set of lysates generated for each treatment timepoint from independent cell culture batches (U251MG human GBM line). Supplementary file3 (PDF 1967 KB) [file 11060_2025_5089_MOESM3_ESM.pdf]

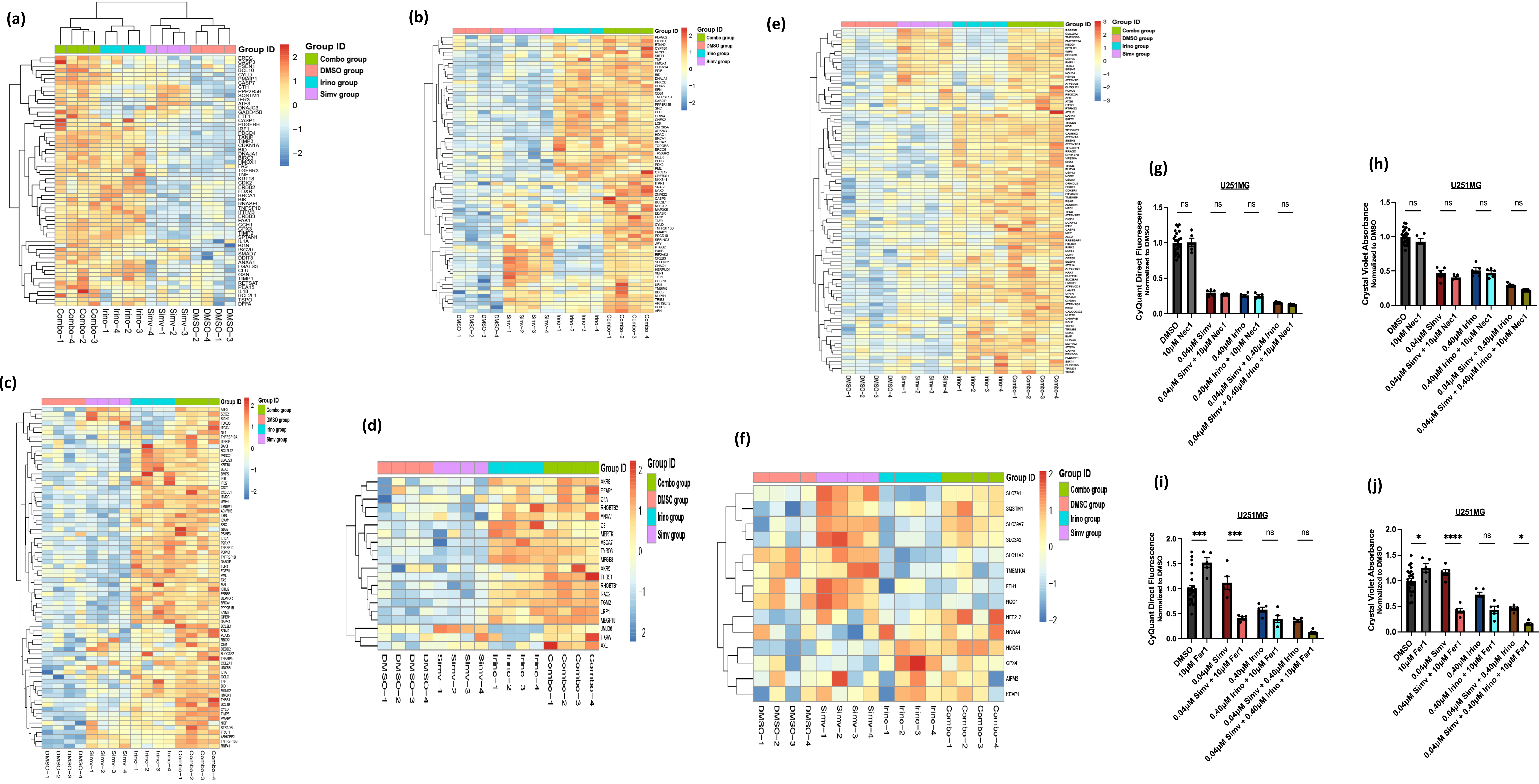

Supplement: Supplementary file 4 — Fig. S4: Simvastatin + irinotecan combination treatment upregulates gene signatures associated with multiple cell death pathways. Gene expression heatmaps showing differentially expressed genes related to (a) Apoptosis pathway, (b) Intrinsic apoptotic signaling pathway, (c) Extrinsic apoptotic signaling pathway, (d) Apoptotic cell clearance pathway, (e) Autophagy pathway, (f) Ferroptosis pathway. (g) CyQuant and (h) Crystal violet quantification of viability loss with simvastatin, irinotecan, simvastatin + irinotecan with/without 10 uM necrostatin1 (necroptosis inhibitor) after 5 days of treatment; (i) CyQuant and (j) Crystal violet quantification of viability loss with simvastatin, irinotecan, simvastatin + irinotecan with/without 10 uM ferrostatin1 (ferroptosis inhibitor) after 5 days of treatment. Data are the result of n ≥ 4 biological replicates per treatment group (U251MG human GBM line). Supplementary file4 (PDF 1146 KB) [file 11060_2025_5089_MOESM4_ESM.pdf]

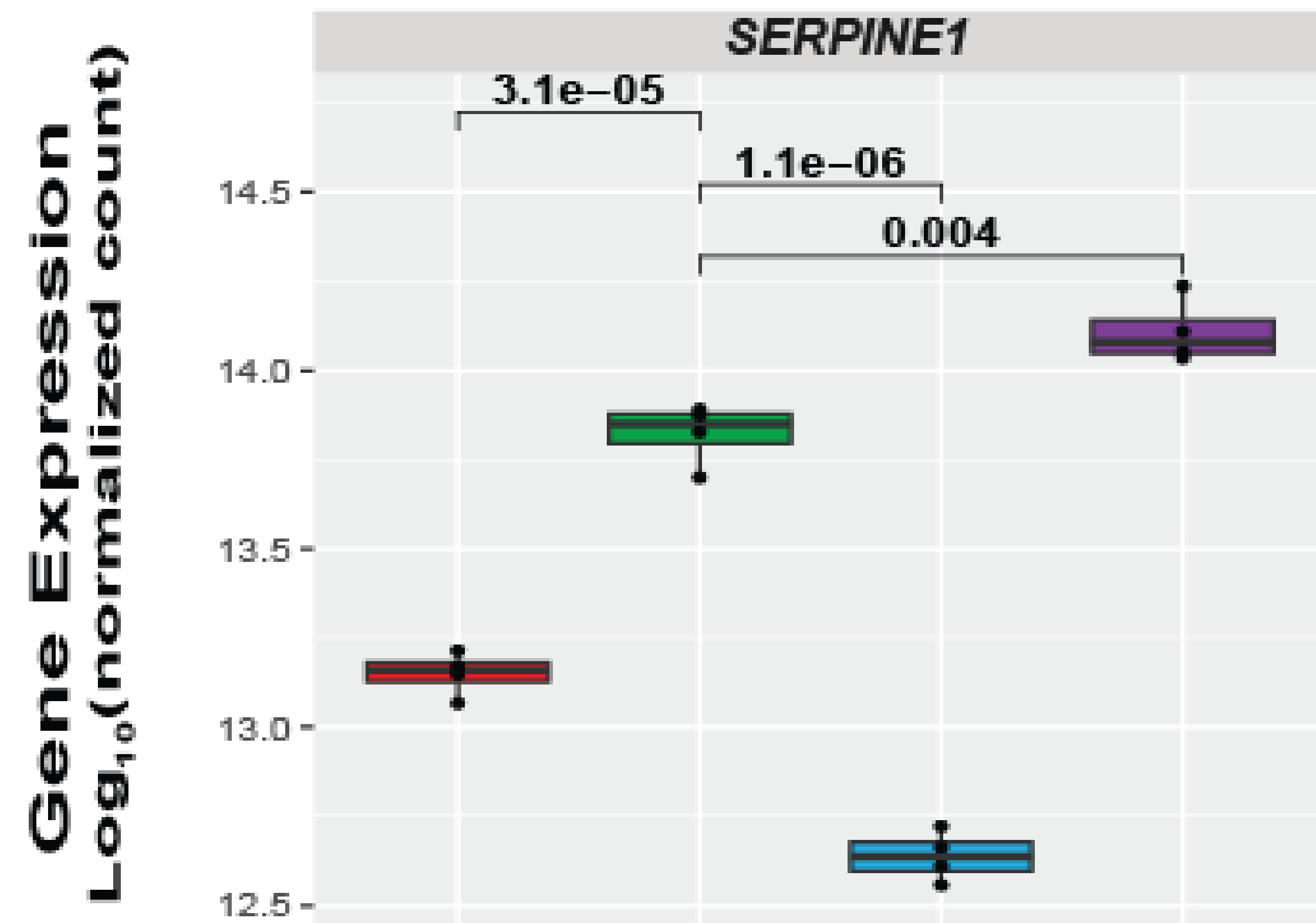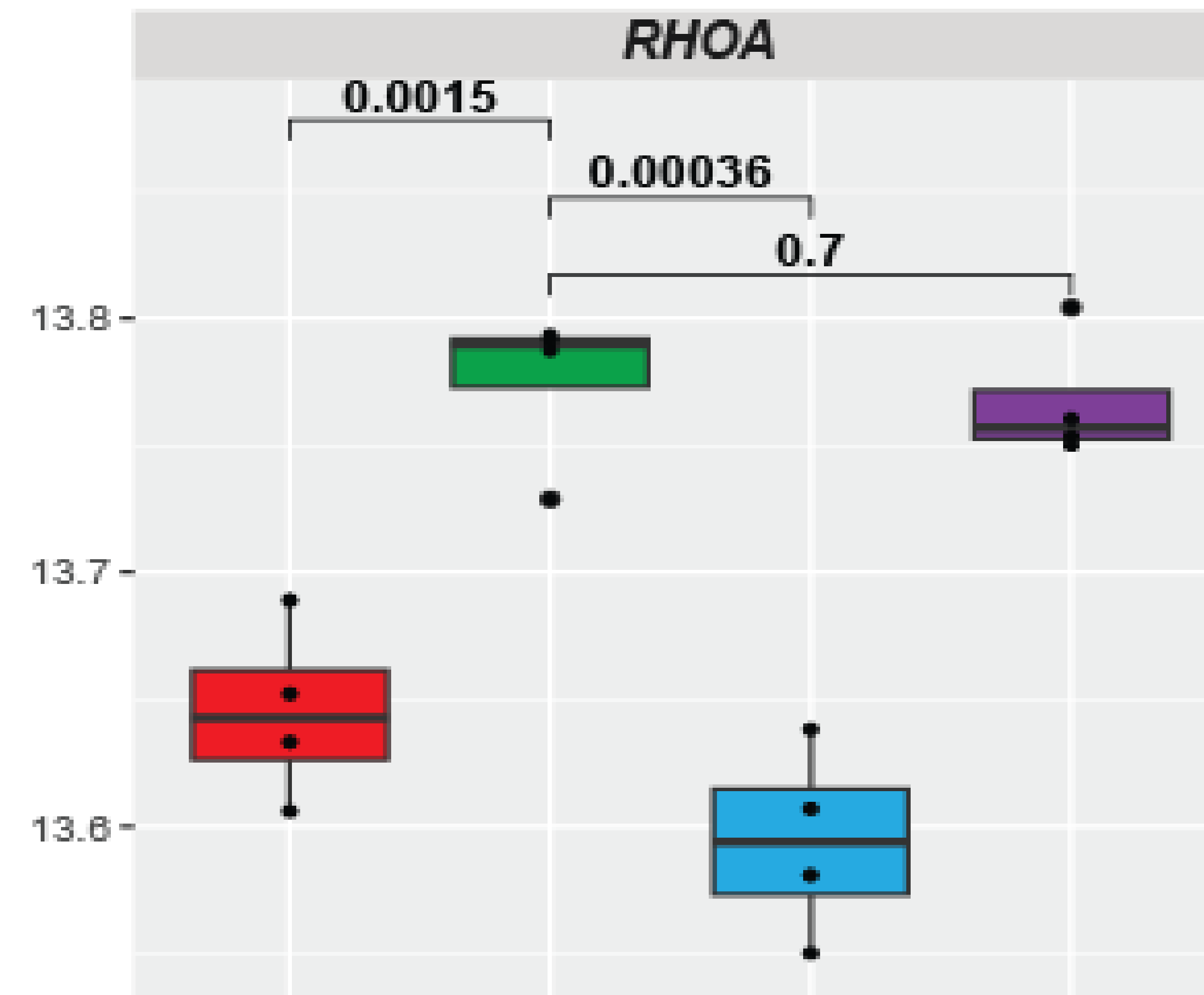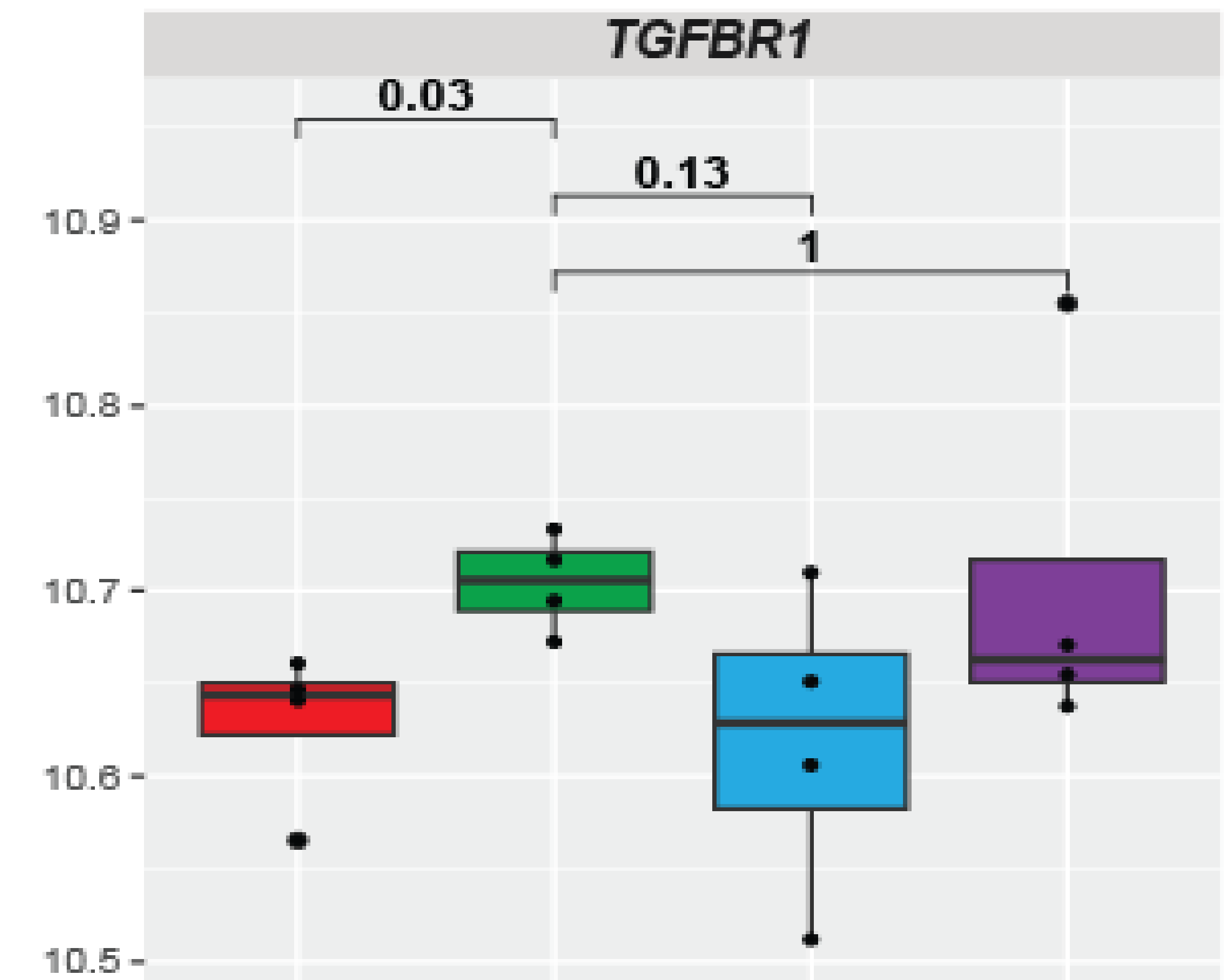

**Group ID**

- Combo\_group
- DMSO\_group
- Irino\_group
- Simv\_group

Supplement: Supplementary file 5 — Fig. S5: Irinotecan alone and in combination with simvastatin decreases gene expression of TGF-β pathway family members. Bulk RNA-Seq boxplots depicting gene expression of selected TGF-β pathway family members (SERPINE1, RHOA, TGFβR1) in DMSO vs. simvastatin single-agent (Simv) vs. irinotecan single-agent (Irino) vs. simvastatin + irinotecan combination (Combo) treated groups at 48 h post-treatment. All data are n = 4 biological replicates per group (U251MG human GBM line). Adjusted p-values in each treatment condition compared to DMSO are shown within the gene expression boxplots. Supplementary file5 (PDF 34 KB) [file 11060_2025_5089_MOESM5_ESM.pdf]
